# Comparative bioactivity evaluation and metabolic profiling of different parts of *Duhaldea nervosa* based on GC-MS and LC-MS

**DOI:** 10.3389/fnut.2023.1301715

**Published:** 2023-12-08

**Authors:** Qian Zhao, Yuan Li, Si Li, Xiaofeng He, Rui Gu

**Affiliations:** ^1^School of Pharmacy, Chengdu University of Traditional Chinese Medicine, Chengdu, China; ^2^School of Ethnic Medicine, Chengdu University of Traditional Chinese Medicine, Chengdu, China; ^3^State Key Laboratory of Southwestern Chinese Medicine Resources, School of Pharmacy, Chengdu University of Traditional Chinese Medicine, Chengdu, China

**Keywords:** antioxidants, novel foods, *Duhaldea nervosa*, metabolomics, structural identification

## Abstract

*Duhaldea nervosa* (Wallich ex Candolle) Anderberg has been widely used as medicine and food additive in China for a long history. Its roots, known as Xiaoheiyao, are the mainly used medicinal part, while the other tissues of *D. nervosa* are ignored as non-medicinal parts despite their high biomass, resulting in a huge waste of resources. To mine and expand the medicinal values of different parts of *D. nervosa*, metabolic analysis by GC/LC-MS and bioactivity evaluation were performed. Based on the antioxidant activity and correlation analysis, a metabolite-related network was constructed. A total of 45 volatile and 174 non-volatile compounds were identified. Among them, caffeoylquinic acids and derivatives were more abundant in roots and flowers, while coumaroyltartaric acids and derivatives were mainly present in stems and leaves. By multivariate analysis, 13 volatile and 37 non-volatile differential metabolites were found, respectively. In the bioactivity evaluation of different parts, the order of antioxidant capacity was flowers > roots > leaves or stems. The flowers showed the highest FRAP value (354.47 μM TE/g DW) and the lowest IC_50_ values in the DPPH (0.06 mg/mL) and ABTS (0.19 mg/mL) assay, while higher inhibitory activity against α-glucosidase was exhibited by flowers and leaves. This study first established the similarities and differences of phytochemicals and bioactivities in *D. nervosa*, providing a scientific basis for developing non-medicinal parts and guiding the clinical application of this medicinal and edible herb.

## 1 Introduction

*Duhaldea nervosa* (Wallich ex Candolle) Anderberg is a medicinal and edible herb of Asteraceae family, and is mainly distributed in the Southwest of China. The roots of *D. nervosa*, known as Xiaoheiyao, have been long used as a traditional Chinese medicine (TCM) to treat stomachache and relieve rheumatism (1). Furthermore, the roots of *D. nervosa* have also been popularly used in cooking as food additives, such as being stewed with chicken in a soup (it is believed to relieve dizziness), which was officially approved as a new food material by the Ministry of Health of PR China in 2010 (2).

With the extensive development of new plant-derived functional foods and dietary supplements, many edible plant sources that are rich in antioxidants (such as phenolic compounds) have begun attracting the public attention (3). Studies have shown that natural plants are rich in antioxidants, which are effective, easy to be absorbed, and almost have no side effect. Therefore, screening antioxidants with potential therapeutic effects from natural plants has a promising prospect.

Previous studies have demonstrated that *D. nervosa* contains steroids, terpenoids, flavonoids, polysaccharides, and phenolic acids (4), which have a wide range of bioactivities including anti-inflammatory, neuroprotective, antioxidant, hepatoprotective, and anticancer properties (5). Some studies have revealed that the root extracts protect HepG2 cells from H_2_O_2_-induced oxidative stress by increasing the expression of Nrf2 and related antioxidant enzymes (6).

However, the focus of previous studies was only on the roots of *D. nervosa*, while the medicinal potential of other parts was unknown. Consequently, to highlight the medicinal, nutritional and edible importance of this medicinal and edible herb, it is of great significance to further develop the application potential of *D. nervosa* and fully explore the components and bioactivity in different parts of *D. nervosa*.

Metabolomics is aimed at identifying differences in total metabolite fingerprints, which has significant advantages in the detection of chemical components that are significantly different from each other. The UPLC-Q-Orbitrap HRMS (ultra-high-pressure liquid chromatography coupled with Orbitrap high-resolution mass spectrometry) based metabolomics is featured by its high sensitivity, high resolution, and specificity, And has been widely used to identify the major different chemical components in various plant samples (7). To date, there is no report about the metabolite comparison of the different parts of *D. nervosa*.

In this study, the metabolic profiles of roots (the traditional medicinal part) and other non-medicinal parts (including flowers, stems and leaves) of *D. nervosa* were analyzed by gas chromatography-mass spectrometer (GC-MS) and UPLC-Q-Orbitrap HRMS. The differential metabolites between the traditional medicinal part and other non-medicinal parts of *D. nervosa* based on chemometrics were then screened. In addition, the DPPH, ABTS, FRAP and α-glucosidase bioassay of different parts were performed, and by correlation analysis the relationship between differential metabolites and the biological effects of different parts were determined. These results provided guidance for clinical application and quality control of the waste parts of *D. nervosa* as potential medicinal sources.

## 2 Materials and methods

### 2.1 Chemicals and reagents

*Duhaldea nervosa* (Wallich ex Candolle) Anderberg was collected in Panzhihua, Sichuan, China. The whole plant was dried and parted into roots, stems, leaves, and flowers, separately.

MS grade acetonitrile, formic acid and methanol were obtained from Fisher Scientific (Fair Lawn, NJ, United States). Distilled water was purchased from the A. S. Watson Group (Hong Kong, China). 2-Chloro-l-phenylalanine, 2,2′-azino-bis (3-ethylbenzthiazoline-6-sulphonic acid) diammonium salt (ABTS) and 2,2-diphenyl-1-picrylhydrazyl (DPPH) and 2,4,6-tri(2-pyridinyl)-1,3,5-triazine (TPTZ) were purchased from Shanghai Yuanye Biotechnology Co., Ltd. (Shanghai, China). α-Glucosidase was purchased from Sigma (St. Louis, MO, United States). All standard compounds used in this study (3,5-dicaffeoylquinic acid, 3,4-dicaffeoylquinic acid, 4,5-dicaffeoylquinic acid, 3-caffeoylquinic acid, 4-caffeoylquinic acid, 5-caffeoylquinic acid, scopoletin and kaempferol) with a purity >98% were obtained from Chengdu Ruifensi Biotechnology Co, Ltd. (Chengdu, China).

### 2.2 UPLC-Q-Orbitrap HRMS analysis

#### 2.2.1 Sample preparation

All dried samples from different parts of *D. nervosa* were finely ground and sieved, yielding approximately 0.5 g of pulverized powder. Ultrasonic extraction was performed by immersing the powder in 25 mL of methanol/water (7:3, v/v) for 1 h (8). Subsequently, the extracts were centrifuged at 12,000 rpm for 10 min, and then the supernatant was filtered through a 0.22 μm membrane for the UPLC-Q-Orbitrap HRMS analysis.

#### 2.2.2 UPLC-Q-Orbitrap HRMS

Vanquish UPLC system coupled with Q Exactive Orbitrap high-resolution MS (Thermo Fisher Scientific, Waltham, United States) was used for metabolite analysis. Instrument and data acquisition were performed by Xcalibur 4.1 software. Sample separation was performed on a Thermo Scientific Accucore^™^ C_18_ column (100 mm × 3 mm, 2.6 μm) at 25°C. The flow rate was 0.3 mL min^−1^, and the injection volume was 3 μL. The multi-step gradient program was beneficial to improve the separation efficiency, and the elution condition was conducted according to the references with some changes (9), the comparisons of chromatograms obtained under different experimental conditions were shown in Supplementary Figure S1, we decided to use the elution condition of Supplementary Figure S1C because it has a better separation. The mobile phase consisted of 0.1% (v/v) formic acid in water (A) and acetonitrile (B) with a gradient programme: 0–12 min, 2–30% B; 12–25 min, 30% B; 25–35 min, 30–32% B; 35–40 min, 32–34% B; 40–45 min, 34–70% B; 45–50 min, 70–95% B, 50–55 min, 95–2%. The source parameters were set as follows: spray voltage, 3.5 kV (+) /3.0 kV (−); capillary temperature, 320°C; heater temperature, 350°C; sheath gas flow rate, 35 arb; aux gas flow rate, 10 arb. The Orbitrap analyser scanned over a mass-to-charge ratio (*m*/*z*) range of *m*/*z* 100 to 1,500 Da with a resolution of 35,000 in full scan MS^1^ and a resolution of 17,500 in dd-MS2. The mixed normalised collision energy was set at 20, 40 and 60 V. Quality control (QC) samples were injected every 3 samples throughout the run to monitor system stability.

#### 2.2.3 Identification of compounds

Using Compound Discover v3.1 software, the raw MS data collected by UPLC-Q-Orbitrap HRMS were first screened and combined with online databases including mzCloud and mzVault (Thermo Fisher Scientific, Waltham, United States) and self-built databases, and unknown metabolites were identified based on concordance between MS^1^ and MS^2^.

### 2.3 GC-MS analysis

#### 2.3.1 Sample preparation

Powdered samples (5 g) were sonicated in 50 mL of *n*-hexane for 40 min, then the supernatant was dried under a nitrogen stream to 1 mL and filtered through a 0.22 μm membrane to obtain the sample solutions. All solutions were stored at 4°C prior to analysis.

#### 2.3.2 GC-MS conditions and compounds identification

Analysis was conducted on an Agilent Technologies 7890A GC system and an Agilent Technologies 5975C inert MSD equipped with triple-axis detector (Agilent, United States). Sample separation was operated on a HP-INNOWax capillary column (30 m × 250 μm × 0.25 μm, Agilent, United States). The helium flow rate was controlled at 1 mL/min. A 10°C/min ramp was set from an initial temperature of 50°C to 150°C, then to 180°C at 5°C/min, and then to 250°C at 3°C/min, for a total of 50 min. A sample volume of 1 μL was injected at a split ratio of 10:1. Spectra were recorded in the full scan range (from 35 to 1,000 *m*/*z*) with the EI source of positive ion mode, source temperature of 230°C, and quadrupole temperature of 150°C. MS Workstation v6.9.3 (Agilent, United States) was used for instrument control and data processing. Compounds were searched and identified by the NIST14. L database (NIST, United States).

### 2.4 Statistical analysis

The UPLC-Q-Orbitrap HRMS data were analyzed using Compound Discover software, which produced a matrix of features containing MS, retention time and peak area through peak extraction, deconvolution, peak alignment and other operations. The data of GC-MS were processed by peak alignment and gap filling, and a feature matrix was generated through peak area normalization. The two matrices of features were then imported into SIMCA 14.1 (Umetrics, Sweden) for principal component analysis (PCA), hierarchical cluster analysis (HCA), and orthogonal partial least squares discriminant analysis (OPLS-DA). R2(*cum*) and Q2(*cum*) values were used to validate the model. R2 represents the ability to explain the original data, and Q2 represents the predictive ability of the model. Data were analyzed using a combination of variable importance in the projection values (VIP, VIP > 1), fold changes (FC, FC > 2 or FC < 0.5) and *p*-values (*p* < 0.05) from the *t*-test for potential differential metabolites. Heatmap visualization was performed using MetaboAnalyst 5.0.

### 2.5 Evaluation of bioactivity

The supernatants were diluted to different concentrations for the DPPH, ABTS, FRAP (10) and α-glucosidase (11) bioactivity assay with slight modifications based on literatures. All experiments were carried out independently and repeated more than three times, and the experimental data were expressed as mean ± standard deviation.

## 3 Results and discussion

### 3.1 UPLC-Q-Orbitrap HRMS

Compounds were identified by self-built and online databases (mzCloud and mzVault). The self-built database of 169 compounds was constructed from the phytochemical and pharmacological literature by searching the SciFinder, ChemSpider, Google Scholar and CNKI databases, which contained detailed information such as compound name, molecular formula and structural formula. Moreover, compound identification was conducted using MS/MS fragmentation patterns compiled from reference material along with considerations of retention time, characteristic ions, signal intensity, and relevant literature.

The hydroxycinnamic acids (mainly including caffeoylquinic acids, coumaroyltartaric acids and their derivatives) were more easily to be detected in the negative ion mode, whereas the flavonoids were detected in the positive ion mode. A total of 174 compounds, including 54 caffeoylquinic acids and derivatives, 13 feruloylquinic acids and derivatives, 8 *p*-coumaroylquinic acids and derivatives, 6 caffeoyltartaric acids and derivatives, 29 other hydroxycinnamic acids and derivatives, 16 hydroxybenzoic acids derivatives, 36 flavonoids, 5 coumarins, 4 lignan derivatives, and 3 other compounds were identified from the roots, flowers, stems and leaves of *D. nervosa*. The total ion chromatograms (TICs) of samples are shown in Supplementary Figures S2, S3. The specific fragment information is shown in Table 1.

**Table 1:** Metabolites in roots (R), flowers (F), stems (S), and leaves (L) of *D. nervosa* identified by UPLC-Q-Orbitrap HRMS.

No.	Retention time (min)	Experimental (*m*/*z*)	Diff. (ppm)	Molecular formula	Fragments (*m*/*z*)	Tentative Identification	Source				Ref
							R	F	S	L	
*Hydroxycinnamic derivatives*											
*Caffeoylquinic acids and derivatives*											
1	5.11	353.0287 [M-H]^−^	−2.16	C_16_H_18_O_9_	191.0558; 179.0343; 135.0442	1-CQA	−	+	+	+	(12)
2	7.26	353.0880 [M-H]^−^	0.18	C_16_H_18_O_9_	191.0557; 173.0450	Cis-3-CQA	+	+	+	+	(12)
3	7.47	353.0519 [M-H]^−^	−2.34	C_16_H_18_O_9_	191.0556; 173.0084	Tran-3-CQA	+	+	+	+	(12)
4	7.66	353.0538 [M-H]^−^	2.87	C_16_H_18_O_9_	191.0557; 179.0348; 135.0443	Cis-4-CQA	+	+	+	+	(12)
5	7.64	353.1819 [M-H]^−^	0.52	C_16_H_18_O_9_	193.9559; 191.0558; 173.0088	Tran-5-CQA	−	+	+	+	(12)
6	10.58	353.0874 [M-H]^−^	−1.2	C_16_H_18_O_9_	191.0557; 179.0345; 161.0237	Tran-4-CQA	+	+	+	+	(12)
7	11.13	353.0875 [M-H]^−^	−1.01	C_16_H_18_O_9_	191.0345; 173.0450; 135.0444	Cis-5-CQA	+	+	+	+	(12)
8	4.56	515.1414 [M-H]^−^	−1.33	C_22_H_28_O_14_	353.0891; 191.0556; 179.0345	CQA-3′-hexoside	+	+	+	+	(13)
9	5.55	515.1416 [M-H]^−^	−0.8	C_22_H_28_O_14_	353.0901; 323.0777; 191.0556; 179.0347; 161.0237	CQA-4′-hexoside	+	+	+	+	(13)
10	7.92	515.1913 [M-H]^−^	3.35	C_25_H_24_O_12_	353.0879; 191.0555; 179.0343	1,3-DiCQA	+	+	+	+	(8)
11	10.57	515.1190 [M-H]^−^	−0.92	C_25_H_24_O_12_	353.0878; 191.0556; 179.0345; 135.0444	1,4-DiCQA	+	+	+	+	(8)
12	10.84	515.1193 [M-H]^−^	−0.37	C_25_H_24_O_12_	353.0879; 191.0557; 179.0346; 135.0444	3,4-DiCQA	−	+	−	+	(8)
13	12.03	515.1766 [M-H]^−^	−3.4	C_25_H_24_O_12_	353.0877; 191.0555; 179.0344; 173.0447; 135.0443	1,5-DiCQA	+	−	+	+	(14)
14	12.32	515.1195 [M-H]^−^	0.58	C_25_H_24_O_12_	353.0877; 191.0554; 179.0343; 161.0235; 135.0443	3,5-DiCQA	+	+	+	+	(14)
15	13.06	515.2134 [M-H]^−^	3.07	C_25_H_24_O_12_	353.0881; 191.0558; 173.0450; 179.0337	4,5-DiCQA	+	−	+	+	(12)
16	13.10	515.2165 [M-H]^−^	3.17	C_25_H_24_O_12_	353.0876; 191.0554; 179.0344	Tran-4-Cis5-DiCQA	−	+	+	+	(12)
17	14.28	515.1775 [M-H]^−^	−1.67	C_25_H_24_O_12_	353.0880; 191.0555; 179.0341; 173.0454	DiCQAI	−	−	+	+	(14)
18	14.19	515.2129 [M-H]^−^	3.19	C_25_H_24_O_12_	353.0887; 191.0558; 179.0346; 173.0449; 135.0444	DiCQAII	+	−	−	+	(14)
19	14.50	515.2137 [M-H]^−^	−2.02	C_25_H_24_O_12_	353.0888; 191.0555; 179.0341; 173.0446	DiCQAIII	+	−	+	+	(14)
20	8.40	677.1732 [M-H]^−^	4.93	C_34_H_30_O_15_	515.1425; 353.0883; 191.0556; 179.0343	TriCQAI	−	+	−	−	(12)
21	8.63	677.1733 [M-H]^−^	−0.86	C_34_H_30_O_15_	515.1408; 353.0891; 179.0345	TriCQAII	+	+	+	+	(12)
22	9.16	677.1721 [M-H]^−^	−2.08	C_34_H_30_O_15_	515.1333; 353.0869; 179.0341	TriCQAIII	+	+	+	−	(12)
23	9.32	677.1722 [M-H]^−^	4.45	C_34_H_30_O_15_	515.1411; 353.0878; 191.0557	1,4,5-TriCQA	+	+	+	−	(9)
24	9.54	677.1722 [M-H]^−^	3.84	C_34_H_30_O_15_	515.1326; 353.0876; 191.0557; 179.0345	1,3,5-TriCQA	+	+	+	−	(9)
25	9.97	677.1732 [M-H]^−^	−0.85	C_31_H_34_O_17_	515.1216; 353.0876; 191.0557; 173.0451; 179.0344	DiCQA-hexosideVI	+	+	+	+	(9)
26	10.13	677.1725 [M-H]^−^	4.02	C_31_H_34_O_17_	515.1398; 353.0693; 179.0344; 173.0451	DiCQA-hexosideV	+	+	+	+	(9)
27	10.82	677.1514 [M-H]^−^	−2.07	C_31_H_34_O_17_	515.1167; 179.0344; 135.0442	DiCQA-hexosideI	+	+	+	+	(9)
28	11.36	677.1514 [M-H]^−^	−1.93	C_31_H_34_O_17_	515.1192; 471.1298; 335.0779	DiCQA-hexosideII	+	+	+	+	(9)
29	11.60	677.1515 [M-H]^−^	−1.4	C_31_H_34_O_17_	515.1181; 335.0769; 179.0345	DiCQA-hexosideIII	−	+	+	+	(9)
30	11.73	677.1519 [M-H]^−^	−2.45	C_31_H_34_O_17_	515.1211; 335.0770; 161.0237	DiCQA-hexosideIV	+	+	−	+	(9)
31	12.81	677.1521 [M-H]^−^	−0.79	C_34_H_30_O_15_	515.1191; 353.0879; 191.0556	3,4,5-TriCQA	−	−	+	−	(12)
32	13.68	677.1514 [M-H]^−^	−1.8	C_34_H_30_O_15_	515.1208; 353.0873; 191.0556; 179.0343	1,3,4-TriCQA	+	+	+	+	(12)
33	8.27	839.2263 [M-H]^−^	−1.28	C_37_H_43_O_22_	515.1408; 191.0556; 179.0344	DiCQA-dihexoside	−	+	−	−	(15)
34	4.92	707.1832 [M-H]^−^	−1.42	C_32_H_35_O_18_	353.0879; 191.0556; 179.0343	3-CQA dimer	+	+	+	−	(15)
35	5.08	707.1834 [M-H]^−^	4.51	C_32_H_35_O_18_	515.7155; 191.0555	5-CQA dimer	−	−	+	+	(15)
36	6.10	707.1826 [M-H]^−^	−2.34	C_32_H_35_O_18_	353.0879; 191.0556; 179.0334	CQA dimerII	+	+	+	−	(15)
37	6.46	707.1835 [M-H]^−^	−1.07	C_32_H_35_O_18_	353.0875; 191.0556; 179.0344; 173.0449	4-CQA dimer	+	+	+	+	(15)
38	14.97	707.1630 [M-H]^−^	−0.57	C_32_H_35_O_18_	353.0884; 193.0499; 191.0555; 179.0344	CQA dimerI	+	+	+	+	(15)
39	7.68	335.0777 [M-H]^−^	−3.33	C_16_H_16_O_8_	179.0345; 161.0237; 135.0444	3-CQL	+	+	+	+	(9)
40	7.80	335.1255 [M-H]^−^	1.76	C_16_H_16_O_8_	179.0343; 161.0233; 135.0445	1-CQL	−	−	+	+	(9)
41	13.45	497.0733 [M-H]^−^	−1.23	C_25_H_21_O_11_	335.0385; 161.0234,	DiCQL	+	−	+	+	(9)
42	9.21	497.3343 [M-H]^−^	−0.23	C_22_H_25_O_13_	353.7760, 303.6761	CQL-hexosideI	+	+	+	+	(9)
43	12.54	497.1098 [M-H]^−^	−0.2	C_22_H_25_O_13_	335.0772; 179.0342; 135.0444	CQL-hexosideII	+	+	+	+	(9)
44	3.58	371.0625 [M-H]^−^	−2.15	C_16_H_20_O_10_	353.0870; 209.0299; 191.0557; 173.0444	2H,3CQA	−	+	+	+	(16)
45	4.34	371.0985 [M-H]^−^	1.24	C_16_H_20_O_10_	353.1272; 209.0301; 191.0190	2H,5CQA	−	+	+	+	(16)
46	3.44	371.0987 [M-H]^−^	−2.75	C_16_H_20_O_10_	209.0664; 191.0555; 173.0445; 135.0442	5H,5CQA	+	+	+	+	(16)
47	7.47	533.1309 [M-H]^−^	−0.94	C_25_H_26_O_13_	371.0974; 353.0894; 191.0556; 179.0343; 135.0443	3C,4HCQAI	+	+	+	+	(12)
48	7.68	533.1312 [M-H]^−^	−1.66	C_25_H_26_O_13_	371.0977; 353.0877; 191.0557; 179.0344; 135.0443	3C,4HCQAII	+	+	+	+	(12)
49	7.92	533.2610 [M-H]^−^	−1.34	C_25_H_26_O_13_	353.0887; 191.0445; 173.0448; 171.0991; 135.0443	3HC,4CQA-hexoside	+	+	+	+	(12)
50	8.33	533.1305 [M-H]^−^	3.37	C_25_H_26_O_13_	371.0996; 353.0879; 335.0762; 191.0559; 173.0452	3C,5HCQA	+	+	+	+	(12)
51	8.18	533.1309 [M-H]^−^	−1	C_25_H_26_O_13_	371.0996; 353.0879; 335.0762; 191.0559; 173.0452	3HC,4CQA	+	+	+	+	(16)
52	9.53	533.0936 [M-H]^−^	−2.61	C_25_H_26_O_13_	371.0619; 209.0299; 191.0193	3HC,5CQA	+	+	+	+	(16)
53	10.01	533.1659 [M-H]^−^	0.66	C_25_H_26_O_13_	371.0610; 323.0773; 179.0341; 161.0237; 135.0443	4C,5HCQA	+	+	+	−	(16)
54	11.74	533.2234 [M-H]^−^	−3.57	C_25_H_26_O_13_	371.0623; 353.0493; 191.0191	4HC,5CQA	−	−	+	+	(16)
*Feruloylquinic acids and derivatives*											
55	6.12	367.0675 [M-H]^−^	−1.62	C_17_H_20_O_9_	193.0504; 173.0453; 134.0367	Cis-3-FQA	+	+	+	+	(17)
56	7.80	367.0130 [M-H]^−^	−2.92	C_17_H_20_O_9_	193.0495; 191.0556; 134.0364	Tran-3-FQA	+	+	+	+	(17)
57	7.99	367.1037 [M-H]^−^	0.45	C_17_H_20_O_9_	193.0505; 191.0557; 173.0448	Cis-4-FQA	+	+	+	+	(17)
58	11.66	367.1399 [M-H]^−^	3.74	C_17_H_20_O_9_	163.0758; 135.0808	Cis-5-FQA	+	+	+	+	(17)
59	11.72	367.1185 [M-H]^−^	−4.2	C_17_H_20_O_9_	163.0758; 135.0708	Tran-5-FQA	+	+	+	+	(17)
60	12.07	367.1034 [M-H]^−^	−0.25	C_17_H_20_O_9_	193.0503; 191.0559; 134.0365	Tran-4-FQA	+	+	+	−	(17)
61	11.59	529.1927 [M-H]^−^	−2.45	C_26_H_26_O_12_	353.0886; 349.0913; 335.0769; 193.0502; 179.0346	3F,4CQA	+	+	+	+	(12)
62	12.07	529.1351 [M-H]^−^	−2.44	C_26_H_26_O_12_	367.1037; 193.0501; 134.0365	3F,5CQA	+	+	+	+	(9)
63	12.21	529.2275 [M-H]^−^	2.71	C_26_H_26_O_12_	367.1024; 353.0872; 191.0556; 161.0232; 135.0439	1C,5FQA	+	−	−	+	(12)
64	12.47	529.1356 [M-H]^−^	−1.87	C_26_H_26_O_12_	367.1026; 353.0878; 173.0447; 135.0443	3C,5FQA	+	+	+	+	(12)
65	12.68	529.1561 [M-H]^−^	0.91	C_26_H_26_O_12_	353.0878; 191.0556; 179.0342; 173.0447; 135.0443	1C,4FQA	+	+	+	+	(12)
66	13.01	529.2660 [M-H]^−^	−2.1	C_26_H_26_O_12_	353.0877; 191.0556; 179.0344; 173.0449; 135.0443	4C,5FQA	+	+	+	+	(12)
67	14.32	529.1936 [M-H]^−^	1.82	C_23_H_30_O_14_	367.1036; 353.6503; 161.0237	CFQA-hexoside	+	+	+	+	(12)
*p-coumaroylquinic acids and derivatives*											
68	5.86	337.0933 [M-H]^−^	1.34	C_16_H_18_O_8_	191.0558; 163.0396	3-*p*CoQA	+	+	+	+	(18)
69	8.47	337.0932 [M-H]^−^	0.75	C_16_H_18_O_8_	191.0557; 173.0448; 163.0395	5-*p*CoQA	+	+	+	+	(18)
70	11.51	499.1247 [M-H]^−^	0.86	C_25_H_24_O_11_	337.0936; 191.0561; 163.0396	3-*p*Co,5CQA	+	+	−	−	(9)
71	11.77	499.1249 [M-H]^−^	−2.12	C_25_H_24_O_11_	353.0881; 337.0929; 191.0557; 179.0344; 163.0394	3C, 5-*p*CoQA	+	+	+	+	(8)
72	12.11	499.1247 [M-H]^−^	−2.59	C_25_H_24_O_11_	353.0868; 337.0918; 191.0556; 179.0347; 163.0396; 135.0444	4-*p*Co,5CQA	−	+	+	−	(9)
73	12.32	499.1253 [M-H]^−^	−1.33	C_25_H_24_O_11_	337.0934; 353.0884; 191.0562; 173.0450; 163.0394	Cis-4-*p*Co,5CQA	+	+	+	+	(9)
74	12.36	499.1645 [M-H]^−^	0.76	C_25_H_24_O_11_	353.7108; 337.0920; 179.0340; 173.0449	4C, 5-*p*CoQA	+	−	−	−	(9)
75	13.20	661.1574 [M-H]^−^	−0.58	C_35_H_32_O_14_	499.1226; 353.0876; 191.0556; 179.0344; 135.0443	*p*CoDiCQA	+	+	+	+	(8)
*Caffeoyltartaric acids and derivatives*											
76	4.70	311.0411 [M-H]^−^	0.79	C_13_H_12_O_9_	179.0345; 149.0085; 135.0444	CTAI	−	−	+	+	(19)
77	9.26	311.0406 [M-H]^−^	−0.58	C_13_H_12_O_9_	179.0345; 149.0084; 135.0443	CTAII	+	+	+	+	(19)
78	9.27	473.0723 [M-H]^−^	−3.32	C_22_H_18_O_12_	311.0407; 293.0307; 179.0344; 149.0084; 135.0443	DiCTAI	+	+	+	+	(18)
79	9.68	473.0722 [M-H]^−^	−3.57	C_22_H_18_O_12_	311.0409; 179.0344; 149.0084; 135.0443	DiCTAII	−	+	+	+	(18)
80	10.71	473.0726 [M-H]^−^	−2.68	C_22_H_18_O_12_	311.0408; 293.0305; 219.0292; 179.0343; 149.0083; 135.0442	DiCTAIII	−	+	+	−	(18)
81	10.68	457.1352 [M-H]^−^	−2.72	C_20_H_26_O_12_	295.0458; 293.0312; 179.0344; 163.0395	*p*CoCTA	+	−	+	+	(20)
*Other hydroxycinnamic acids and derivatives*											
82	10.57	179.0335 [M-H]^−^	−3.04	C_9_H_8_O_4_	135.0443; 109.1621; 107.0493	Caffeic acid	+	+	+	+	(15)
83	14.34	207.0662 [M-H]^−^	0.99	C_11_H_12_O_4_	179.0343; 161.0238; 135.0444	Dimethylcaffeic acid	+	+	+	+	(15)
84	5.96	341.0879 [M-H]^−^	−3.6	C_15_H_18_O_9_	281. 0668; 221.0449; 179.0346; 161.0238; 135.0443	Caffeoyl-*O*-hexoside	+	+	+	+	(16)
85	7.71	341.1246 [M-H]^−^	−2.2	C_15_H_18_O_9_	179.0708; 135.0807	CA-hexosideI	+	+	+	+	(12)
86	9.28	341.0667 [M-H]^−^	−3.98	C_15_H_18_O_9_	179.0345; 161.0238; 135.0444	CA-hexosideII	+	+	+	+	(12)
87	9.81	341.1242 [M-H]^−^	−3.91	C_15_H_18_O_9_	179.0708; 161.0452; 135.0807	CA-hexosideIII	+	+	+	+	(12)
88	5.72	343.1040 [M-H]^−^	1.63	C_15_H_20_O_9_	181.0500; 135.0439	Dihydrocaffeic acid-hexosideI	+	+	+	+	(18)
89	6.17	343.1034 [M-H]^−^	0.09	C_15_H_20_O_9_	181.0500; 179.0347; 135.0443	Dihydrocaffeic acid-hexosideII	+	+	+	+	(18)
90	7.91	295.0463 [M-H]^−^	1.43	C_13_H_12_O_8_	179.0342; 135.0445; 133.0134; 115.0028	Caffeoylmalic acid	+	+	+	+	(18)
91	7.11	297.0620 [M-H]^−^	−3.02	C_13_H_14_O_8_	135.0292; 113.0600	Caffeoylthreonate	+	+	+	+	(18)
92	5.87	357.1169 [M-H]^−^	−4.1	C_15_H_18_O_10_	195.0505; 151.0755; 135.0446	Caffeoylgluconic acidI	+	+	+	+	(18)
93	6.68	357.1195 [M-H]^−^	0.98	C_15_H_18_O_10_	195.0558; 177.0548; 165.0551	Caffeoylgluconic acidII	+	+	+	−	(18)
94	7.02	695.1472 [M-H]^−^	4.64	C_33_H_28_O_17_	533.1143; 371.1300; 209.0299; 179.0348	Tricaffeoyl citric acidII	+	+	+	+	(15)
95	10.80	695.1253 [M-H]^−^	−2.23	C_33_H_28_O_17_	533.0938; 371.0618; 209.0298; 191.0192	Tricaffeoyl citric acidI	+	+	+	+	(15)
96	1.79	193.0706 [M + H]^+^	−2.23	C_7_H_12_O_6_	176.0109; 127.0392; 85.0285	Quinic acid	+	+	+	+	(21)
97	4.08	353.1459 [M-H]^−^	−1.85	C_13_H_22_O_11_	191.0560; 179.0346; 135.0447	QA-hexosideI	+	+	+	+	(9)
98	4.92	353.0879 [M-H]^−^	0.15	C_13_H_22_O_11_	191.0556; 179.0344; 135.0444	QA-hexosideII	+	+	+	+	(9)
99	7.02	193.0503 [M-H]^−^	−1.39	C_10_H_10_O4	165.0550; 135.0444; 121.02867	Ferulic acid	+	+	+	+	(22)
100	12.31	517.1261 [M-H]^−^	3.54	C_22_H_30_O_14_	353.0862; 269.0458; 191.0564; 173.0450; 179.0339	FA-dihexoside	+	+	+	+	(22)
101	6.32	325.0574 [M-H]^−^	−1.33	C_15_H_18_O_8_	163.0242; 145.0135; 119.0497	*p*-Coumaric acid-hexosideI	+	+	+	+	(18)
102	9.96	325.1295 [M-H]^−^	−3.36	C_15_H_18_O_8_	163.0759; 135.0807; 119.0342	*p*-Coumaric acid-hexosideII	+	+	+	+	(18)
103	5.89	327.0724 [M-H]^−^	−3.26	C_15_H_20_O_8_	179.0345; 165.0399; 135.0446	Dihydro-*p*Coumaric acid-hexosideI	+	+	+	+	(23)
104	7.88	327.1088 [M-H]^−^	−2.87	C_15_H_20_O_8_	207.1050; 165.0551; 163.0390	Dihydrocoumaroyl-*O*-hexosideI	+	−	+	+	(23)
105	11.61	327.1450 [M-H]^−^	0.51	C_15_H_20_O_8_	207.1016; 165.0913; 121.0286	Dihydrocoumaroyl-*O*-hexosideII	+	+	+	+	(23)
106	6.18	295.0460 [M-H]^−^	0.11	C_13_H_12_O_8_	163.0393; 119.0493	*p*-Coumaroyltartaric acid	+	+	+	+	(18)
107	8.88	487.1275 [M-H]^−^	−3.23	C_21_H_28_O_13_	343.1395; 325.1282; 191.0555; 133.0664	*p*-Coumaric acid-dihexosideI	−	−	+	+	(23)
108	9.67	487.1822 [M-H]^−^	2.93	C_21_H_28_O_13_	343.1401; 325.1306; 163.0754	*p*-Coumaric acid-dihexosideII	+	+	+	+	(23)
109	8.80	223.0610 [M-H]^−^	−0.78	C_11_H_12_O_5_	176.0107; 148.0158	Sinapinic acid	+	+	+	+	(18)
110	6.18	385.1141 [M-H]^−^	−3.52	C_17_H_22_O_10_	223.0610; 208.0373; 179.0709	Sinapic acid-hexoside	+	+	+	+	(18)
*Hydroxybenzoic acids and derivatives*											
111	2.01	149.0085 [M-H]^−^	−4.7	C_4_H_6_O_6_	121.0284; 87.0078	Tartaric acid	+	+	+	+	(18)
112	4.35	153.0188 [M-H]^−^	−3.54	C_7_H_6_O_4_	109.0286; 91.0181	Gentisic acid	+	+	+	+	(24)
113	5.15	315.0724 [M-H]^−a^	0.73	C_13_H_16_O_9_	153.0185; 109.0285	Protocatechuic acid-4-*O*-hexoside	+	+	+	+	(21)
114	4.61	315.0724 [M-H]^−^	0.88	C_13_H_16_O_9_	153.0551; 123.0444; 109.0287	Protocatecheuic acid 3-*O*-glucoside	+	+	+	+	(21)
115	4.51	153.0550 [M-H]^−^	−4.66	C_7_H_6_O_4_	109.0286	Protocatechuic acid	+	−	+	+	(21)
116	5.61	137.0237 [M-H]^−^	0.38	C_7_H_6_O_3_	137.0236; 109.0286; 93.0336	Protocatechualdehyde	+	+	+	+	(21)
117	6.06	167.0342 [M-H]^−^	0.36	C_8_H_8_O_4_	123.0442	Vallic acid	+	−	+	+	(25)
118	18.70	169.0864 [M-H]^−^	−3.36	C_7_H_6_O_5_	125.0963	Gallic acid	+	+	+	+	(25)
119	2.02	173.0086 [M-H]^−^	−3.47	C_7_H_10_O_5_	111.0080; 85.0286	Shikimic acid	+	+	+	+	(25)
120	4.94	197.0815 [M-H]^−^	−2.14	C_9_H_10_O_5_	151.0757; 123.0443	Syringic acid	+	+	+	−	(19)
121	3.51	331.0675 [M-H]^−^	−2.67	C_13_H_16_O_10_	313.0564; 169.0152; 168.006	Gallic acid-hexoside	+	+	+	+	(25)
122	4.64	359.0986 [M-H]^−^	−3.68	C_15_H_20_O_10_	197.0451; 182.0215; 153.0550; 138.0315	Syringic acid-hexoside	+	+	+	+	(19)
123	2.80	137.0459 [M + H]^+^	0.84	C_7_H_6_O_3_	110.0355; 95.0859	Hydroxybenzoic acid	+	+	+	+	(18)
124	5.81	299.0775 [M-H]^−^	−3.56	C_13_H_16_O_8_	137.0236; 93.0336	Hydroxybenzoic acid-hexoside	+	+	+	+	(18)
125	13.44	435.0933 [M-H]^−^	0.05	C_20_H_20_O_11_	315.0723; 152.0108; 137.0236	Hydroxybenzoyl-*_O_*-dihydroxybenzoic acid-hexoside	+	+	+	+	(18)
126	12.62	451.0887 [M-H]^−^	−1.93	C_20_H_20_O_12_	331.0674; 313.0569; 168.0057	Hydroxybenzoyl gallic acid-hexoside	+	+	+	+	(18)
*Flavonoids*											
127	9.57	303.0498 [M + H]^+^	−0.38	C_15_H_10_O_7_	257.0439; 229.0492; 183.0288; 165.0184; 153.0183	Quercetin	+	+	+	+	(24)
128	10.14	505.0993 [M-H]^−a^	1.08	C_23_H_22_O_13_	300.0276; 301.0353; 271.0250; 255.0295; 178.9981; 151.0028	Quercetin 3-*O*-acetylgalactoside	+	+	+	+	(26)
129	10.21	505.1358 [M-H]^−^	1.21	C_23_H_22_O_13_	301.0347; 300.0283; 271.0254; 255.0300; 151.0028	Quercetin 3-*O*-acetylglycoside	+	+	+	+	(26)
130	9.20	463.2181 [M-H]^−^	−0.72	C_21_H_20_O_12_	301.0351; 300.0276; 271.0246; 255.0301; 151.0030	Quercetin 3-*O*-glucoside	+	+	+	−	(26)
131	11.39	493.1340 [M + H]^+a^	0.16	C_23_H_24_O_12_	331.0810; 316.0576; 301.0341	Dimethylquercetin-7-*O*-hexoside	−	+	+	+	(27)
132	10.10	479.1180 [M + H]^+^	−0.6	C_22_H_22_O_12_	317.0683; 302.0418	3-*O*-methylquercetin-*O*-hexoside	+	+	+	+	(27)
133	11.47	479.1187 [M + H]^+^	0.71	C_22_H_22_O_12_	317.0655; 302.0421	Methylquercetin-hexoside	−	+	+	+	(27)
134	11.39	331.0808 [M + H]^+^	−1.22	C_17_H_14_O_7_	316.0577; 301.0338; 273.0369	7,4-dimethylquercetin	−	+	+	−	(27)
135	16.38	331.0812 [M + H]^+^	0.3	C_17_H_14_O_7_	316.0575; 315.0490; 301.0338	3,5-dimethylquercetin	−	+	+	−	(27)
136	14.23	639.1369 [M-H]^−^	1.11	C_27_H_28_O_18_	463.1056; 283.0231	Quercetin hexose-glucuronide	−	+	+	−	(18)
137	10.97	301.1185 [M + H]^+^	0.54	C_16_H_12_O_6_	286.0468; 285.0406; 258.0516	Isokaempferide	−	−	+	−	(28)
138	15.99	299.0562 [M-H]^−^	0.12	C_16_H_12_O_6_	286.0469; 258.0521	Kaempferide	+	+	+	+	(28)
139	8.41	579.1346 [M + H]^+^	0.32	C_27_H_30_O_14_	299.0547; 271.0602	Kaempferitrin	+	+	+	−	(28)
140	13.48	285.0406 [M-H]^−^	0.11	C_15_H_10_O_6_	257.0461; 267.0300; 199.0396; 151.003	Kaempferol	+	+	+	+	(13)
141	10.73	461.2394 [M-H]^−^	−2.44	C_22_H_22_O_11_	285.0403; 255.0299; 227.0347	Kaempferol-7-*O*-glucuronide	+	+	+	+	(29)
142	9.68	595.1651 [M + H]^+^	−0.74	C_27_H_30_O_15_	449.1067; 287.0546	Kaempferol-hexoside deoxyhexoside	+	+	+	+	(27)
143	9.35	595.1654 [M + H]^+^	−0.27	C_27_H_30_O_15_	449.1073; 287.0547	Kaempferol-3-*O*-rutinoside	+	+	+	+	(27)
144	6.41	447.1878 [M-H]^−^	4.46	C_21_H_20_O_11_	285.0400; 284.0328	Kampferol-3-*O*-glucoside	+	+	+	+	(24)
145	9.69	449.1074 [M + H]^+^	−0.83	C_21_H_20_O_11_	287.0548	Kaempferol-hexoside	+	+	+	+	(27)
146	10.65	271.0596 [M + H]^+^	−1.71	C_15_H_10_O_5_	225.0552; 153.0182; 119.0494	Apigenin	+	+	+	+	(30)
147	9.42	431.1924 [M-H]^−^	2.66	C_21_H_20_O_10_	341.0684; 269.0453	Apigenin 7-*O*-glucoside	−	+	−	+	(30)
148	10.99	447.0919 [M + H]^+^	−0.31	C_21_H_18_O_11_	271.0598; 153.0182	Apigenin 7-*O*-glucuronide	+	+	+	+	(12)
149	12.18	473.1086 [M-H]^−^	−3.5	C_23_H_22_O_11_	268.0378; 269.0456	Apigenin-7-*O*-acetylglucoside	−	+	+	+	(12)
150	10.83	577.0282 [M-H]^−^	−0.77	C_27_H_30_O_14_	269.0455; 268.0377	Apigenin 7-*O*-rutinoside	−	+	+	+	(30)
151	9.50	287.0546 [M + H]^+^	−1.31	C_15_H_10_O_6_	213.0334; 179.0289; 153.0181; 135.0439	Luteolin	+	+	+	+	(18)
152	7.48	447.1877 [M-H]^−^	4.11	C_21_H_20_O_11_	285.0397; 217.0509; 199.0393	Luteolin 7-*O*-hexoside	−	+	+	+	(18)
153	8.49	447.1877 [M-H]^−^	−2.26	C_21_H_20_O_11_	285.0422; 284.0327; 199.9543	Luteolin 4-*O*-hexoside	+	+	+	+	(18)
154	9.87	461.2393 [M-H]^−^	0.15	C_21_H_18_O_12_	327.0490; 285.0404; 217.0501; 151.0031; 133.0287	Luteolin 7-*O*-glucuronide	+	−	−	−	(31)
155	12.66	461.2398 [M-H]^−^	−1.62	C_21_H_18_O_12_	301.1955; 285.0403	Luteolin 7-*O*-glucuronide isomer	+	+	+	+	(31)
156	7.71	489.1039 [M-H]^−^	−2.58	C_23_H_22_O_12_	285.0329; 255.0287	Luteolin-7-*O*-acetylglucuronide	+	+	+	+	(31)
157	8.82	623.1258 [M-H]^−^	−2.09	C_27_H_28_O_17_	447.0934; 285.0405; 151.0032; 133.0290	luteolin *O*-hexuronosyl-*O*-hexoside	−	+	+	+	(16)
158	9.59	449.1090 [M-H]^−^	0.06	C_21_H_22_O_11_	287.0562; 269.1392; 151.0029; 135.0443	Marein	+	+	+	+	(31)
159	11.01	491.1197 [M-H]^−^	−2.33	C_23_H_24_O_12_	287.0564; 151.0029	Acetylmarein	+	−	+	+	(31)
160	7.22	289.1661 [M-H]^−^	−3.41	C_15_H_14_O_6_	245.0819; 109.0288	Catechin	−	+	+	+	(32)
161	15.72	299.0562 [M-H]^−^	0.05	C_16_H_12_O_6_	284.0328; 256.0379; 227.0346; 212.0473; 165.9907; 136.9873; 117.0339	Hispidulin	+	+	+	+	(33)
162	11.16	301.0701 [M + H]^+^	−1.75	C_16_H_12_O_6_	286.0469; 168.0053	Trihydroxy-methoxyisoflavone	+	+	+	+	(28)
Coumarins											
163	12.07	163.0330 [M + H]^+^	3.33	C_9_H_6_O_3_	145.0284; 135.0441; 117.0337	Hydroxycoumarin	+	+	+	+	(18)
164	4.85	177.0187 [M-H]^−^	−1.79	C_9_H_6_O_4_	149.0238; 133.0282; 105.0001	DihydroxycoumarinI	−	−	−	+	(18)
165	6.65	177.0189 [M-H]^−^	−1.5	C_9_H_6_O_4_	133.0288; 121.0286; 109.6691; 105.0337	DihydroxycoumarinII	+	+	+	+	(18)
166	10.94	193.1587 [M + H]^+^	−0.23	C_10_H_8_O_4_	175.1480; 133.1012; 109.0857	Hydroxy-methoxycoumarin	+	+	+	+	(25)
167	5.12	293.1244 [M-H]^−^	−4.27	C_13_H_10_O_8_	177.0186; 133.0284; 105.0334	Maloyl-dihydroxycoumarin	+	+	+	+	(18)
*Lignan derivatives*											
168	10.44	417.1575 [M-H]^−^	4.81	C_22_H_26_O_8_	387.1094; 181.0502	Syringaresinol	+	+	+	+	(18)
169	10.46	579.2085 [M-H]^−^	−1.93	C_28_H_36_O_13_	417.1556; 402.1319; 387.1087	Syringaresinol-hexoseI	+	+	+	+	(18)
170	12.64	579.2303 [M-H]^−^	−2.48	C_28_H_36_O_13_	417.2452; 399.1371	Syringaresinol-hexoseII	−	+	+	−	(18)
171	11.30	621.2192 [M-H]^−^	−1.49	C_30_H_38_O_14_	417.1555; 402.1317; 387.1082	Syringaresinol-acetylhexose	+	+	+	+	(18)
*Others*											
172	2.49	133.0500 [M-H]^−^	−4.62	C_4_H_6_O_5_	115.0038	Malic acid	+	+	+	+	(25)
173	3.56	191.0195 [M-H]^−^	−1.31	C_6_H_8_O_7_	111.0079; 102.9479	Citric acid	−	+	−	−	(25)
174	6.90	197.0816 [M-H]^−^	−1.82	C_11_H_16_O_3_	179.0704; 161.0601; 133.0651	Loliolide	+	+	+	+	(25)

#### 3.1.1 Hydroxycinnamic acids and derivatives

**Figure 1:**
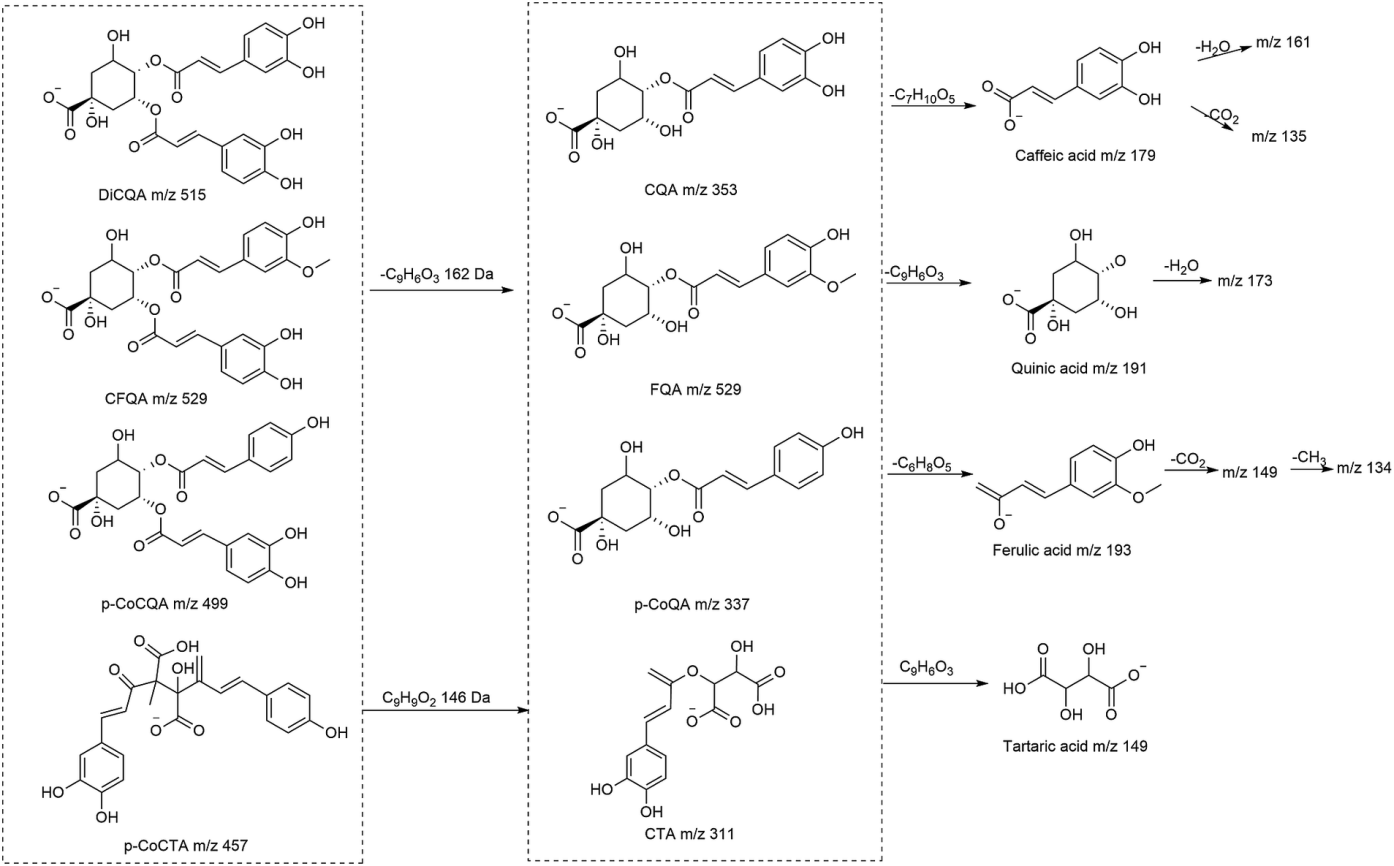
The possible fragmentation pathway of main hydroxycinnamic acids and derivatives.

The hydroxycinnamic acids and derivatives are generally divided into four types, namely caffeoylquinic acids (CQAs), *p*-coumaroylquinic acids (*p*-CoQAs), feruloylquinic acids (FQAs), and caffeoyltartaric acids (CTAs), The structures and explanation of fragmentation behaviors of mass spectra were given in Figure 1, most of which were reported for the first time in the species.

##### 3.1.1.1 Caffeoylquinic acids and derivatives

The caffeoylquinic acids (CQAs) are mainly divided into monoacyl-, diacyl-, and triacyl-quinic acids, CQA-dimers, and their hexosides. Almost all caffeoylquinic acids and their derivatives contain at least caffeic acid-specific MS^2^ fragments at *m*/*z* 135 ([CA-H-CO_2_]^−^), and 161([CA-H-H_2_O]^−^), and at least one quinic acid fragment at *m*/*z* 191 ([QA-H]^−^), and 173 ([QA-H-H_2_O]^−^).

Mono-caffeoylquinic acids included [M-H]^−^ at *m*/*z* 353 (caffeoylquinic acid, CQA), *m*/*z* 335 (caffeoylquinic lactone, CQL), and *m*/*z* 371 (hydroxy dihydro-caffeoylquinic acid, HCQA). The position of the caffeoyl residue in the quinic acid skeleton was related to the relative abundance of base peaks at *m*/*z* 191, 179, 173 and 135. Tran-3-CQA, Tran-5-CQA, and Tran-4-CQA were unambiguously identified by comparing the retention time and MS^2^ data with reference standards (9). Accordingly, compounds **1**, **2**, **4**, and **7** were tentatively presumed as 1-CQA, cis-3-CQA, cis-4-CQA and cis-5-CQA, respectively. Furthermore, the base peak at *m*/*z* 209 [M-caffeoyl]^−^ has a diagnostic value for the HCQA (**44**–**46**). Peaks **39**, **40** were assigned to CQL, deduced from the diagnostic ion at *m*/*z* 161 by losing the lactone and H_2_O moiety (15).

Three common diCQAs subclasses were annotated: di-caffeoylquinic acids (diCQA) at *m*/*z* 515, hydroxydihydro-caffeoyl-caffeolylquinic acids (HC-CQA) at *m*/*z* 533 and caffeoylquinic lactone (diCQL). Peaks (**10**–**19**) have been identified with the same fragment ions at *m*/*z* 515 [M-H]^−^ (C_25_H_24_O_12_), and their MS^2^ spectra at *m*/*z* 353 [M-H-caffeoyl]^−^ suggested they were diCQA. The elution order of the diCQA isomers was 1,3-diCQA > 1,4-diCQA > 3,4-diCQA > 1,5-diCQA > 3,5-diCQA > 4,5-diCQA (3), and compared to the reference standards, they were, respectively, identified. Similarly, peaks **20**–**24** and **31**, **32** had the same quasi-molecular ions [M-H]^−^at *m*/*z* 677 (C_34_H_30_O_15_) that yielded diagnostic product ions at *m*/*z* 515 [M-H-caffeoy]^−^, 353 [M-H-2caffeoy]^−^ and 191 [M-H-3caffeoyl]^−^, which could be inferred as triCQA (13). The HC-CQAs were assigned by the analogy of their MS/MS fragmentation behaviors to those of the diCQAs.

The precursor ion [M-H]^−^ at *m*/*z* 677 (C_31_H_33_O_17_) and the transitions at *m*/*z* 677 → 515 → 353 → 191, indicated the losses of two caffeoyl moieties and a hexoside. Therefore, compound **25**–**30** were preliminarily identified as diCQA-hexosides. In the same way, CQA-hexoside (**8**, **9**), CQL-hexoside (**42**, **43**), and HC, CQA-hexoside (**50**) were identified by the precursor ions at *m*/*z* 353, 497 and 533.

##### 3.1.1.2 Feruloylquinic acids and derivatives

Compounds **55**–**60**, with a precursor ion at *m*/*z* 367, were assigned as mono-FQA. Compounds **55**–**60** were identified as follows: (1) compounds **55** and **56** were identified as 3-FQA by the base peak at *m*/*z* 193 and the “demethylated” ion at *m*/*z* 134 [feruloyl-H-CO_2_-CH_3_]^−^; (2) compounds **58** and **59** were identified as 5-FQA by the base peak at *m*/*z* 163; and (3) with the fragment ion at *m*/*z* 173 (shown as base peak), compounds **57** and **60** were identified as 4-FQA (31). The *cis* or *trans* configuration can be judged from the intensity of these peaks, and the stability of *cis* compounds is more intense (12). Compound **67** had the same quasi-molecular ion [M-H]^−^ at *m*/*z* 529, which was 162 Da more than FQA, indicating that they were the hexosides of FQA.

Peaks **61**–**66** presented the same diagnostic ion at *m*/*z* 529 [M-H]^−^, and by comparison with diCQA and analysis of the characteristic ions at *m*/*z* 367 [M-H-caffeoyl]^−^, 335 [CQA-H-H_2_O]^−^ and 134 [FA-H-CH_3_-CO_2_]^−^, they were finally deduced as caffeoyl-feruloylquinic (CFQA) (9). The fragments at *m*/*z* 349 [FQA-H-H_2_O]^−^ and 335 [CQA-H-H_2_O]^−^ (corresponding to the respective dehydrated ions), and the abundant ion at *m*/*z* 173, assigned 3F,4CQA (**61**) (16). Peak **62** was assigned as 3F,5CQA based on the base peak at *m*/*z* 193 and the abundant ions at *m*/*z* 367 [M-H-caffeoyl]^−^ and 134 as recorded in 3-FQA (9). For **66**, a vicinal 4C, FQA was deduced from the base peak at *m*/*z* 173, and the fragment ions at *m*/*z* 179 and 135 due to the loss of feruloyl.

##### 3.1.1.3 *p*-Coumaroylquinic acids and derivatives

Compounds **68** and **69**, with characteristic ions at *m*/*z* 337 [*p*CoQA-H]^−^ and 163 [*p*CoA-H]^−^, were assigned as *p*CoQA, and the remaining ions were temporarily attributed to fragment series similar to the CQAs (12).

Compounds **70**–**74** showed the deprotonated ion at *m*/*z* 499 [M-H]^−^, 146 Da more than the CQA (an additional sinapoyl residue). The fragment ions at *m*/*z* 191, 163, and 353 (or 337) in the MS^2^ spectrum indicated they were *p*CoCQA. The absence of a base peak at *m*/*z* 173 of compounds **70** and **71** is consistent with 3,5-*p*CoCQA. Thus, compounds **70** and **71** were provisionally designated as 3-*p*Co,5CQA, and 3C,5-*p*CoQA by the base peak and retention time. In addition, the retention time of the 4-substituted *cis*-isomer in the reversed phase column is longer than that of the *trans*-isomer (34). Compounds **72**–**74** were tentatively characterized as 4-*p*Co,5CQA, *cis*-4-*p*Co,5CQA, and 4C,5-*p*CoQA, respectively. The fragment ion at *m*/*z* 661 of compound **75** was similar to the MS^2^ spectrum of *p*CoCQA, so it was tentatively assigned as *p*Co-diCQA (9).

##### 3.1.1.4 Caffeoyltartaric acids and derivatives

Compounds **76** and **77** both presented deprotonated ion at *m*/*z* 311.041 [M-H]^−^, and the fragments of the deprotonated tartaric acid (*m*/*z* 149), caffeic acid (*m*/*z* 179) and the losses of CO_2_ (*m*/*z* 135 [M-H-CA-CO_2_]^−^) showed that they were caffeoyltartaric acids (CTAs). Three compounds **78**–**80** were detected in negative modes at *m*/*z* 473, with the characteristic ion at *m*/*z* 311 [CTA-H]^−^, 293 [M-H-CTA]^−^, 149 [tartaric acid-H]^−^, 179 [CA-H]^−^, and 135 [CA-H-CO_2_]^−^, which were identified as di-caffeoyltartaric acids (diCTA) (18). Compound **81** was detected at *m*/*z* 457 and yielded the MS^2^ ions at *m*/*z* 293, 179 and 163, suggesting to be caffeoyltartaric-*p*-coumaroyl acid (*p*CoCTA) (20).

##### 3.1.1.5 Other hydroxycinnamic acids and hydroxybenzoic acids and their derivatives

On the basis of the fragment patterns by comparison with the reference standards and references, 11 hydroxycinnamic acids (**82**, **83**, **90**, **92**–**96**, **99**, **106**, and **109**) and 8 hydroxybenzoic acids (**111**, **112**, **115**, **117–120**, and **123**) were identified in the extracts, and the losing of neutral molecules [H_2_O (18 Da), CO (28 Da), CO_2_ (44 Da), etc.] were their characteristic fragments. For example, compounds **92** and **93** generated the deprotonated molecule [M-H]^−^ at *m*/*z* 357 and the fragment ions at *m*/*z* 195 [gluconic acid (GA)-H]^−^, 177 [GA-H-H_2_O]^−^ and 165 [GA-H-CH_2_O]^−^, which were the characteristic fragments of caffeoylgluconic acids (34).

According to the MS^2^ spectrum, the fragmentation pattern of hexoside were shown (−162 Da) to identify 14 hydroxycinnamic acids glycosides (**85**–**89**, **92**–**95**, **97**, **98**, **100**–**105**, **118**, **119**, and **110**) and 7 hydroxybenzoic acids glycosides (**113**, **114**, **121**, **122**, and **124**–**126**), and the identification details are shown in Table 1. Besides, MS^2^ spectra of fragment ions resulting from hexose cross cleavages based on the loss of CHOH are as follows: 2Hex (−60 Da), 3Hex (−90 Da) and 4Hex (−120 Da) (16), can distinguish sugar esters and glycosides, which were ascribed as sugar esters, namely caffeoyl-hexoses (**84**) and coumaroyl-hexoses (**104**, **105**).

#### 3.1.2 Flavonoids

The fragmentation features of flavonoids involved the unique neutral removal of acetyl (42 Da), methyl (15 Da), and dimethyl (28 Da) groups, as well as the loss of sugar moieties such as 162, 146, 176, 308, and 324 Da which were, respectively, corresponding to hexose, deoxyhexose, glucuronic acid, rutinoside, and dihexose. Fragment ions, resulting from the neutral losses of CO_2_ (−44 Da), CO (−28 Da), and H_2_O (−18 Da) by the Retro-Diels-Alder (RDA) cleavages of the flavonoid skeleton, were used for the aglycone annotation of quercetin (**127**–**136**), kaempferol (**137**–**145**), apigenin (**146**–**150**) and luteolin (**151**–**157**). In general, flavonoid-*O*-glycosides were the most abundant flavonoids in the extracts. The specific fragment characteristics are presented in Table 1.

**Figure 2:**
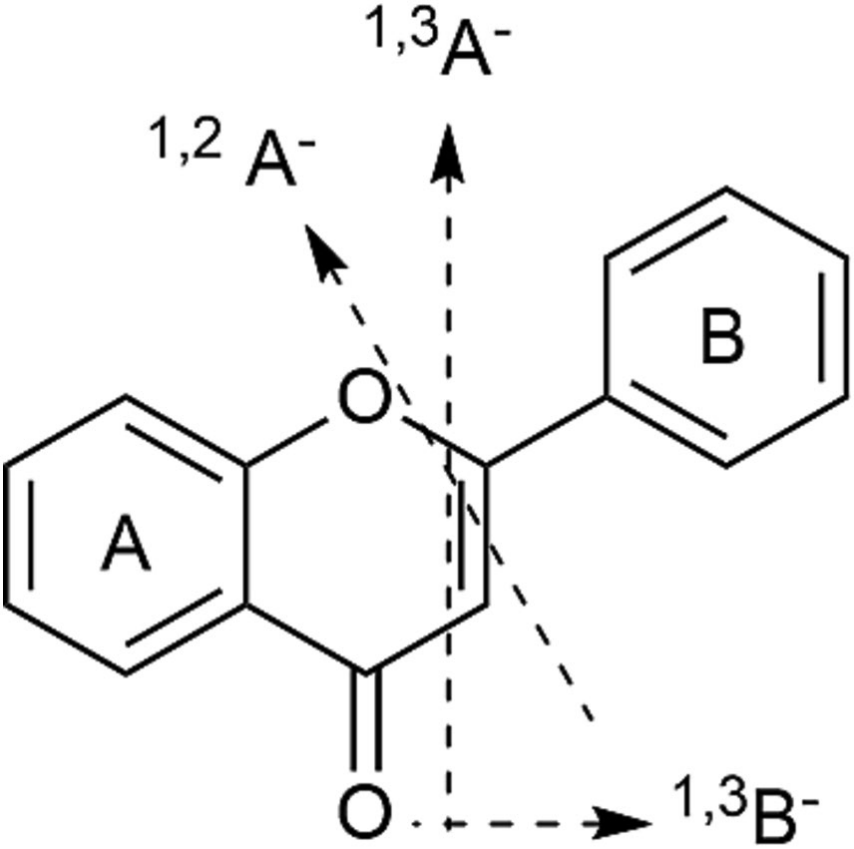
The RDA cleavage mechanisms of the associated flavonoids.

Taking compounds **128**, **129** as example, which presented molecular ions at *m*/*z* 505 (C_23_H_22_O_13_), 301 [M-H-acetyl-glc]^−^ and 255 [M-acetyl-glc-CO-H_2_O]^−^ due to cleavage of the glycosidic bond and loss of neutral ion fragments. The ions at *m*/*z* 179 and 151 were ^1,2^A^−^ and ^1,3^A^−^, obtained by RDA fragmentation (Figure 2 showed the RDA cleavage mechanisms of the associated flavonoids). Additionally, the abundant aglycone radical ion at *m*/*z* 300 was evidence of the 3-*O*-glycosidic linkage. In the positive ion mode, the abundance of the radical aglycone at *m*/*z* 301 and a characteristic ion [M + H-162 Da]^+^ at *m*/*z* 331 revealed the 7-*O*-glycosidic linkage in **131** (18). According to previous studies, the elution order of glycosylated flavonoids at the same position for monosaccharides is galactoside > glycoside on a C_18_ column (18). Thus, compound **127** was assigned as quercetin 3-*O*-acetylgalactoside and **128** was assigned as quercetin 3-*O*-acetylglycoside. In addition, some other flavonoids were annotated as kampferol-3-*O*-glucoside (**144**, diagnostic ion at *m*/*z* 285 [M-H-glc]^−^), apigenin 7-*O*-glucoside (**147**, diagnostic ion at *m*/*z* 269 [M-H-glc]^−^), and luteolin-hexoside (**152**, **153**, diagnostic ions at *m*/*z* 285 [M-H-glc]^−^), 217 [M-H-glc-C_2_H_2_O-C_2_H_2_]^−^ and 199 [M-H-glc-CHO-2CO-H]^−^.

Compounds **136**, **141**, and **154**–**157** were related to the same fragmentation feature and gave characteristic fragment ions at *m*/*z* 463, 285, 271, and 255, respectively, indicating losses of glucuronide moieties (Table 1). Kaempferol-7-*O*-glucuronide (**141**) was deduced from the fragment ions at *m*/*z* 255 [M-glu-CHO-H]^−^ and 227 [M-H-glu-CHO-C_2_H_2_-H]^−^. Compound **154** was assigned as the luteolin 7-*O*-glucuronide based on the fragment ions at *m*/*z* 285 [M-H-glu]^−^ and 217 [M-H-glu-C_2_H_2_O-C_2_H_2_]^−^, as well as RDA ions at *m*/*z* 151 (^1,3^A^−^) and 133 (^1,3^B^−^). Similarly, compound **156** was attributed to luteolin-7-*O*-acetylglucuronide.

In the case of compounds **158** and **159**, based on the fragment ions of [aglycone-H]^−^ at *m*/*z* 287, [aglycone-H-H_2_O]^−^ at *m*/*z* 269, and ^1,3^A^−^ and ^1,3^B^−^ obtained by RDA fragmentation at *m*/*z* 151 and 135, they were tentatively assigned to marein (**158**) and acetylmarein (**159**). Hispidulin (**161**) was determined from the base peak at *m*/*z* 284. To the best of our knowledge, these flavonoids were described for the first time in *D. nervosa*.

#### 3.1.3 Coumarins and lignan derivatives

Lignan derivatives included compounds **168**–**171**. Compound **168**, tentatively identified as syringaresinol (18), was detected at *m*/*z* 417 in negative ion mode with fragment ions at *m*/*z* 399 [M-H-H_2_O]^−^ or 387 [M-H-2CH_3_]^−^. In addition, the loss of fragments of deoxyhexose (−204 Da) and hexose (−162 Da) residues at *m*/*z* 417 could be observed in the MS^2^ of compounds **169**–**171**. Just as the loss of fragments from the syringaresinol at *m*/*z* 402 (-CH_3_), 399 (-H_2_O) or 387 (-2CH_3_), compounds **169**, **170** were then assigned to syringaresinol-hexose and compound **171** was identified as syringaresinol-acetylhexose.

Coumarins derivatives included compounds **163**–**167**. The mass spectrometric fragmentation of coumarin in negative ion mode showed the loss of neutral molecules such as CO (28 Da), CO_2_ (44 Da), and CH_3_ (15 Da) due to high energy collisions. Take compounds **164** and **165** for example, which have the same molecular formula C_9_H_6_O_4_ with the characteristic ion [M-H-CO-H_2_O]^−^ at *m*/*z* 133 and [M-H-2CO]^−^ at *m*/*z* 105, and both were tentatively identified as dihydroxycoumarin (18). All the coumarins and lignans were identified for the first time in the species.

### 3.2 GC-MS analysis

Based on the NIST database and comparison with literature, the GC-MS data were analyzed and identified. There were 45 compounds with match degree greater than 80, the identified compounds could be classified into 9 types, including 11 monoterpenes, 10 sesquiterpenes, 2 diterpenes, 1 triterpene, 6 alkanes, 1 olefin, 12 fatty acids and their esters, 1 ketone, and 1 amide. A total list of compounds, which consists of the formula and adducts, is available in the Supplementary Table S1 and Supplementary Figure S4.

### 3.3 Multivariate statistical analysis of *Duhaldea nervosa*

#### 3.3.1 Distribution of metabolites in different parts

**Figure 3:**
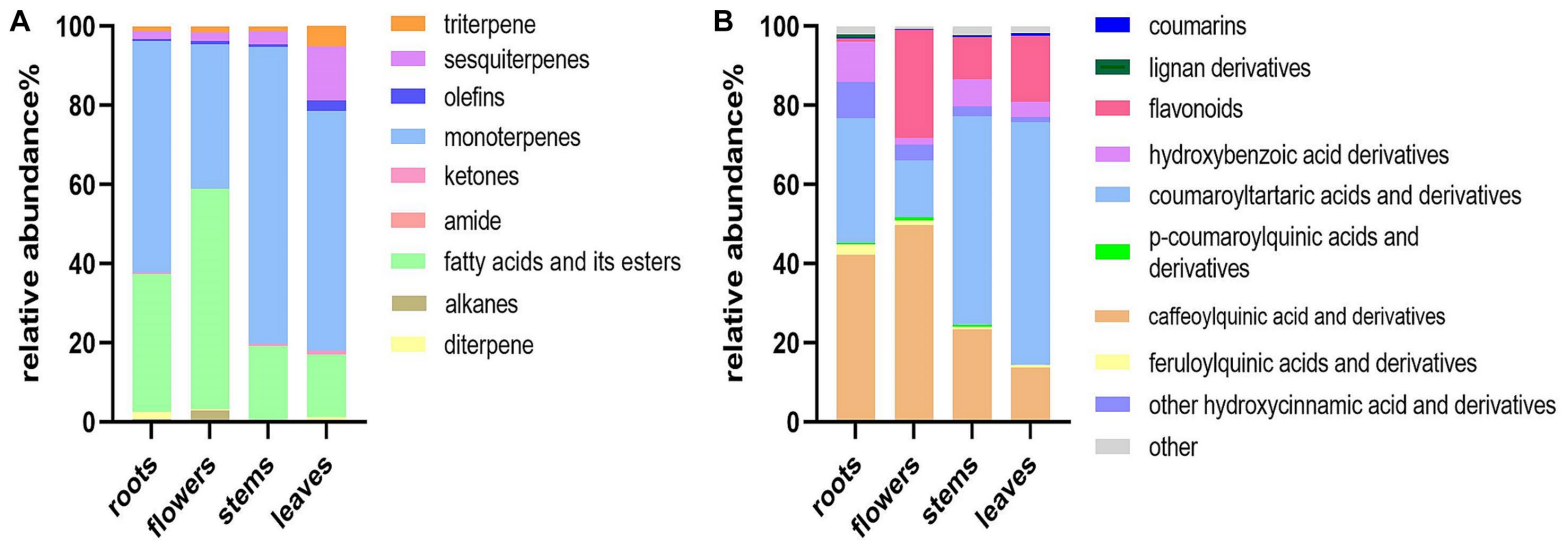
Distribution of the components in different parts of *D. nervosa*. **(A)** Volatile components. **(B)** Non-volatile components.

The relative peak areas of the metabolites were used to construct stack bar graphs of the distribution of metabolites in different parts of *D. nervosa*, as shown in Figure 3. Thymol-based monoterpenes were designated as the main volatile components in roots, flowers, stems, and leaves, with percentages of 58.47, 36.59, 75.00, and 60.52%, respectively. Moreover, the content of fatty acids and their esters in each part is relatively rich. The identified non-volatile compounds were mainly dominated by hydroxycinnamic acids (main including caffeoylquinic acids, coumaroyltartaric acids and their derivatives) in the extracts of different parts, and the contents of the identified total hydroxycinnamic acid and derivatives were roots (85.95%), flowers (70.06%), stems (79.80%) and leaves (77.07%). It is worth noticing that flavonoids were present at low levels in the roots, while hydroxybenzoic acids were more abundant. Flower and roots were richer in caffeoylquinic acids. The content of different parts may be related to the biosynthesis and photosynthesis during flowering, which may affect the synthesis of large amounts of polyphenols (35).

#### 3.3.2 Comparative chemical profiling of different parts of *Duhaldea nervosa*

**Figure 4:**
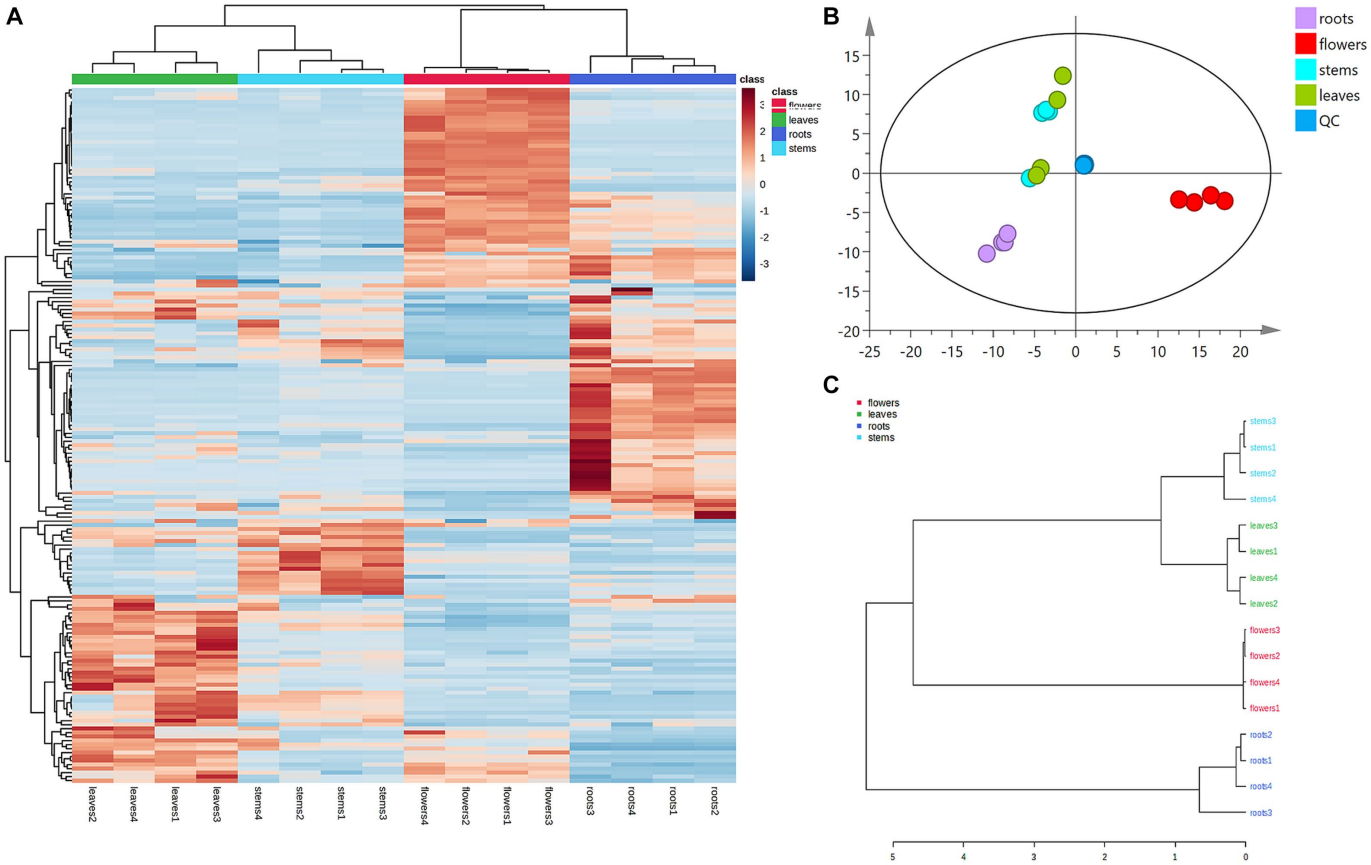
Chemometric analysis for discrimination of non-volatile compounds in different parts (roots, flowers, stems, leaves) of *D. nervosa*. **(A)** Pearson’s rank correlation coefficient of different parts. **(B)** Discriminated by PCA. **(C)** Discriminated by HCA.

**Figure 5:**
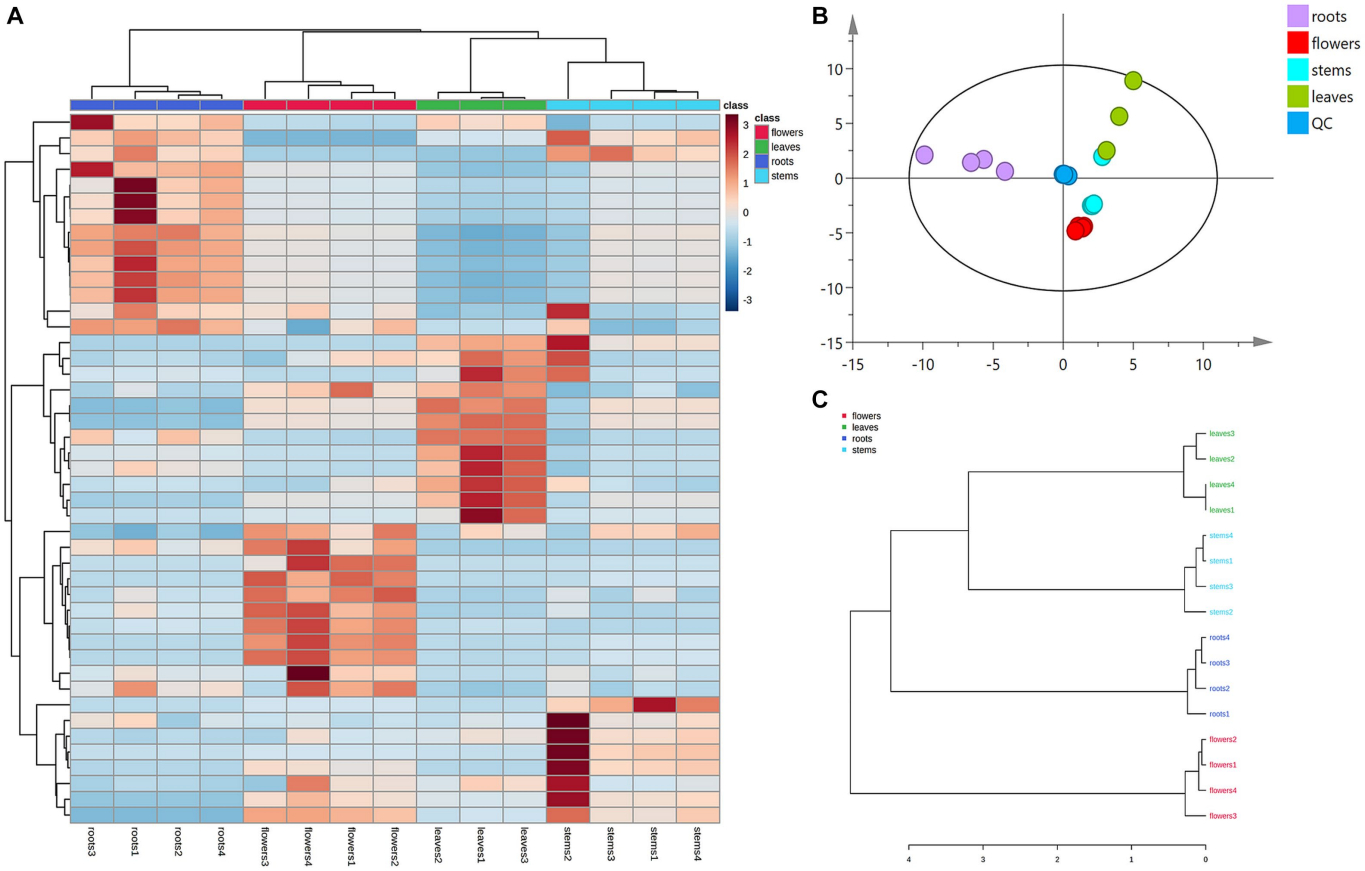
Chemometric analysis for discrimination of volatile compounds in different parts (roots, flowers, stems, leaves) of *D. nervosa*. **(A)** Pearson’s rank correlation coefficient of different parts. **(B)** Discriminated by PCA. **(C)** Discriminated by HCA.

Principal component analysis (PCA) was conducted to classify the different parts of LC-MS (R2X = 0.883) and GC-MS (R2X = 0.964). The samples that fall within 95% of the Hotelling T^2^ ellipse and have no outliers are divided into four groups. As shown in Figures 4B, 5B, the PCA score plots demonstrated that the chemical profile of roots and flowers was significantly different from that of leaves, while the chemical profile of stems was similar to that of leaves. It is significantly indicated by the difference between the medicinal and non-medicinal parts of *D. nervosa*. Organ influence on chemical profiles was more pronounced in roots and flowers, as they showed greater chemical differences. The hierarchical clustering heat map intuitively visualized the degree of difference between chemical profiles in different parts (Figures 4A, 5A). The result of the HCA analysis also clarified it (Figures 4C, 5C), and the dendrogram showed three clusters. They were divided into the flowers cluster, the roots cluster, and finally into two subclusters, one representing the stems and one representing the leaves.

To further clarify the variations between different parts, the non-medicinal parts were compared with roots for OPLS-DA analysis to find marker compounds representing the difference between groups, and heat maps were generated from the relative peak area of different metabolites to visualize the differences in abundance between different parts of *D. nervosa*.

**Figure 6:**
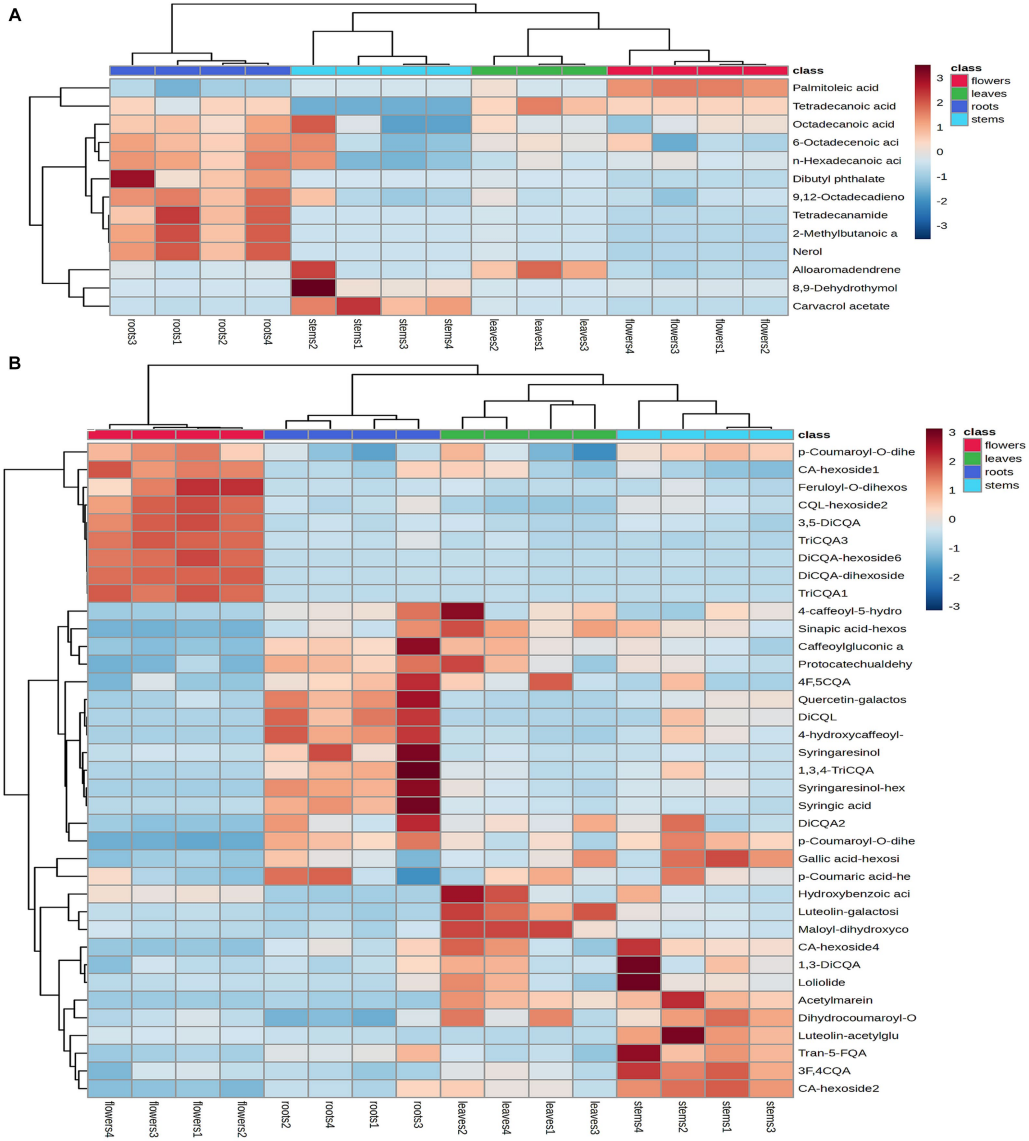
Heatmap of the identified differential metabolites in the volatile compounds **(A)** and non-volatile compounds **(B)**. Heatmap generated by hierarchical Pearson clustering of different metabolites in four subsamples based on mean values, rows represent different metabolites and columns represent samples. Color comparison plot based on relative amounts of metabolites.

Heatmaps were generated from non-repetitive differential compounds (Figure 6A), consisting of 3 monoterpenes, 1 sesquiterpene, and 10 fatty acid and its esters, which were among the 13 differential metabolites identified in various parts. Specifically, the R/F comparative group had 3 differential metabolites, R/S had 2, and R/L had 8 (Supplementary Table S2). The monoterpenes were mainly distributed in stems, while the fatty acids were highly expressed in roots and flowers.

**Figure 7:**
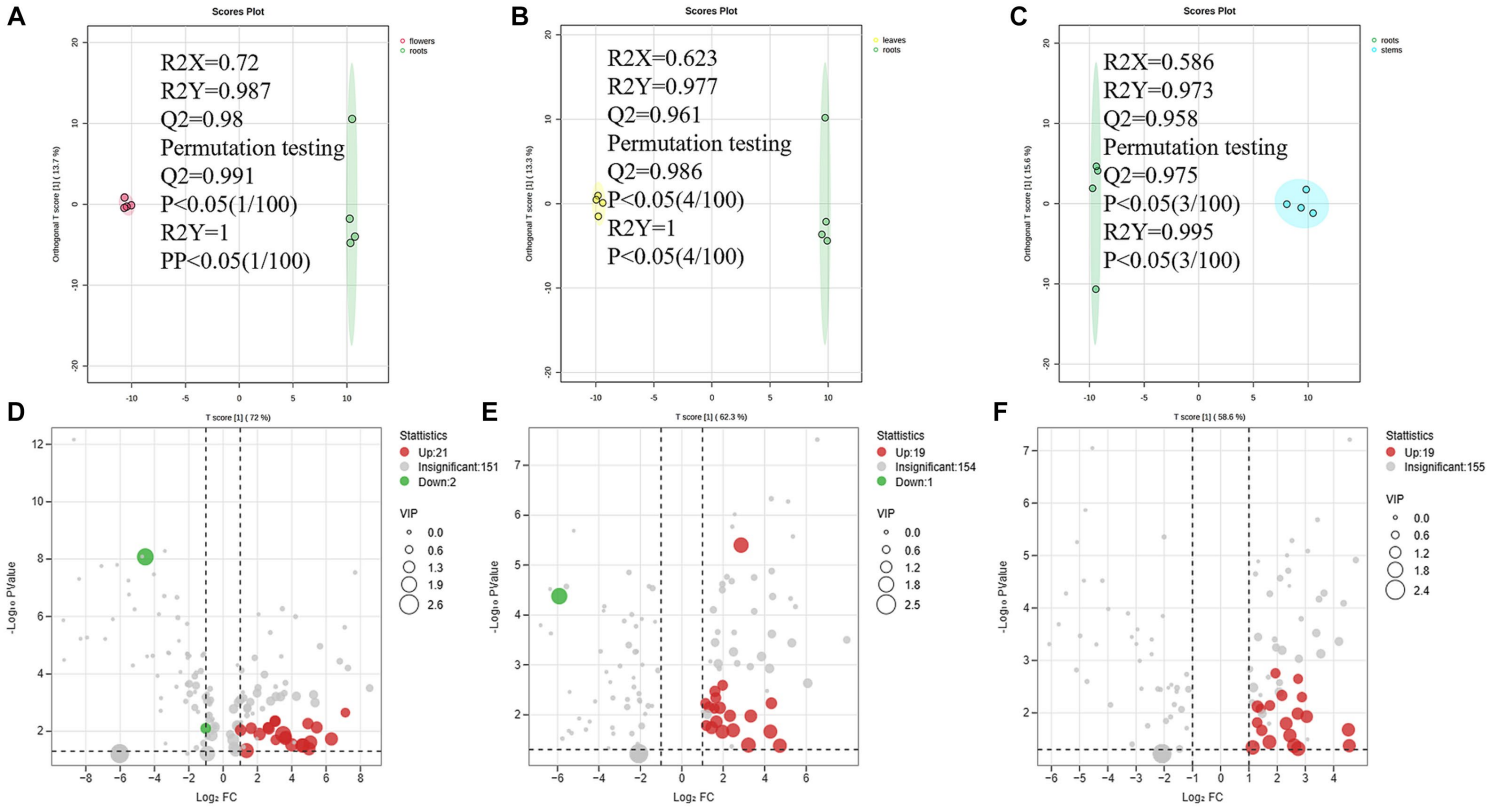
**(A–C)** The OPLS-DA plot between different comparison groups. **(D–F)** The Volcano plots of metabolites of different comparison groups.

A total of 74 differential metabolites were identified using UPLC-Q-Orbitrap HRMS. The result included 23 differential metabolites in the R/F comparative group, 19 in R/S and 18 in R/L (Supplementary Table S3; Figures 7A–F). Thirty-two were non-repetitive differential compounds, including 13 caffeoylquinic acids, 3 feruloylquinic acids, 4 flavonoids, 4 hydroxybenzoic acids, 2 lignans, 1 coumarin, 9 other hydroxycinnamic acids and 1 other. Among them, 37 candidate marker compounds were screened out, which could suggest a remarkable discrimination capability between different parts of *D. nervosa* (Supplementary Table S3 and Figure 6B).

### 3.4 Biological activity of different parts

Excessive production of free radicals can result in oxidative stress, which is strongly linked to the onset of chronic inflammation and degenerative disorders such as cardiovascular diseases, cancers, and autoimmune diseases. Conversely, dietary antioxidants have the potential to diminish the likelihood of encountering these ailments (36). The present study suggested that extracts of *D. nervosa* may serve as natural dietary antioxidants for health promotion, and the roots, flowers, stems and leaves may have significantly different radical scavenging properties. The DPPH, ABTS, and FRAP assays were utilized to assess the antioxidant activity of various parts. Significant variations were observed between the medicinal and non-medicinal sections, with the flowers and roots displaying the highest scavenging activity, followed by the stems, while the leaves exhibited the lowest activity (Table 2). The activity of DPPH was stems < leaves < roots< flowers, and the rise of ABTS and FRAP values were leaves < stems < roots< flowers, which may be explained by the different reaction mechanisms of the ABTS, DPPH, and FRAP.

**Table 2:** Biological activity of different parts, results expressed in crude drug weight (DW) concentration.

Sample	The IC_50_ of antioxidant capacity (mg/mL)			IC_50_ (mg/mL)
	DPPH	ABTS	FRAP (μmol Trolox/g DW ± SD)	α-glucosidase inhibition
Roots	0.3485 ± 0.0405	0.3883 ± 0.0887	174.8048 ± 0.1702	2.6868 ± 0.2005
Flowers	0.0623 ± 0.0485	0.1945 ± 0.0261	354.4689 ± 0.3571	1.7248 ± 0.0721
Stems	1.5110 ± 0.2383	0.9705 ± 0.1462	97.5973 ± 0.4047	4.7905 ± 0.2182
Leaves	1.2443 ± 0.2579	1.0506 ± 0.1132	77.5906 ± 0.3991	1.6628 ± 0.0788
Ascorbic acid	0.0392 ± 0.0331	0.0215 ± 0.0105	/	/
Acarbose	/	/	/	0.0756 ± 0.2043

Data are expressed as mean ± standard deviation (*n* = 3).

**Figure 8:**
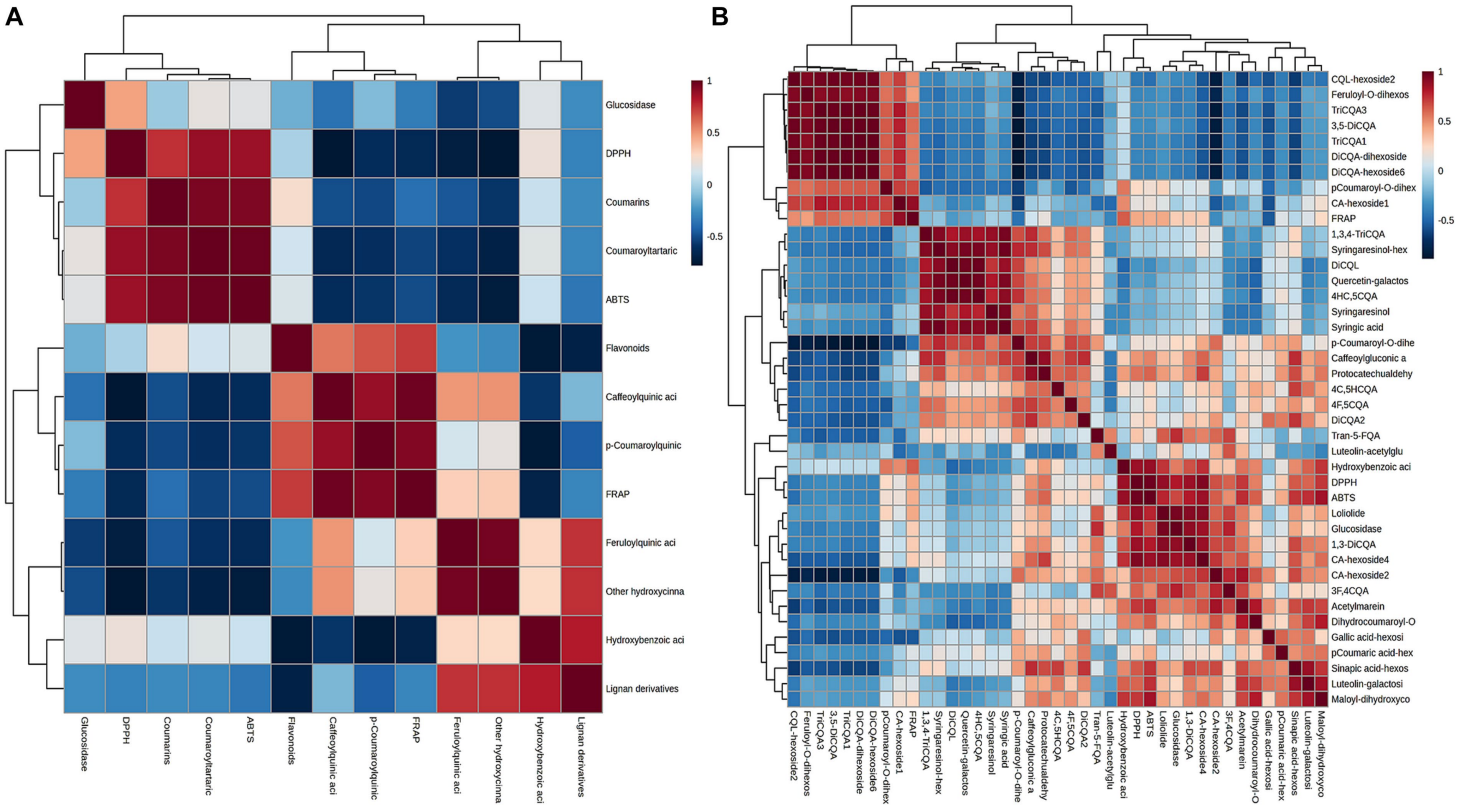
**(A)** Pearson’s correlation matrices between the IC_50_ values of DPPH, ABTS, FRAP and α-glucosidase inhibitory scavenging activities and the different types of metabolites in *D. nervosa*. **(B)** Pearson’s correlation matrices between the IC_50_ values with main differential metabolites.

These findings indicated that the changes in biological activity were pronounced among various plant parts. These variations in bioactivity can be attributed to the differences in the chemical composition of the extracts obtained from different parts. Multivariable models can effectively demonstrate the predictive potential of metabolomic analysis for antioxidant activity and determine which group of metabolites is most responsible for this activity (37). To understand the correlation of constituents, samples were submitted to Pearson’s correlation analysis (Figure 8A). The IC_50_ values of DPPH, ABTS, and FRAP scavenging activities showed a mainly positive correlation with caffeoylquinic acids, feruloylquinic acids, *p*-coumaroylquinic acids, and their derivatives (since IC_50_ values are inverse to antioxidant levels). Furthermore, Pearson’s correlation analysis was used to show the contribution of the main differential metabolites (including diCQA and triCQA) to the antioxidant activity (Figure 8B). CQAs have gained recognition for their ability to perform as antioxidants, reduce inflammation and prevent diabetes. Previous research has shown that the unique molecular structures of these compounds, namely the presence of five active hydroxyl groups and one carboxyl group, contributed to their natural antioxidant properties. The phenolic hydroxyl structure readily reacted with free radicals to produce antioxidant hydrogen radicals that effectively scavenge hydroxyl radicals and superoxide anions, demonstrating their potent antioxidant activity (38). The contents of caffeoylquinic acids and flavonoids were more abundant in the flowers (Figure 3), which might explain the better antioxidant activity of the flowers. In addition, hydroxyl (OH) has a positive effect on the antioxidant properties of phenolic acids, and the relationship between the antioxidant properties of phenolic acids and the number of hydroxyl groups was listed as follows: trihydric phenolic acid > dihydroxyl > mono-hydroxyl (39), and the highest level of correlation between the main metabolites and the antioxidant activity was observed in triCQAs (Figure 6B), which were the most abundant in the flowers and roots.

α-Glucosidase inhibitors slowed the release of glucose from dietary carbohydrates, helping to lower postprandial blood glucose levels, and slow the development of diabetes (40). In our work, the hypoglycaemic activities of different parts were assessed by α-glucosidase inhibition assay. It was found that the flowers and leaves extracts exhibited more potent inhibitory activity than roots extracts (as medicinal parts) (Table 2). Particularly, apart from the quinic acids, the IC_50_ values of α-glucosidase inhibition showed a positive relationship with the amount of flavonoids. Flowers and leaves are richer in flavonoids compared to roots and stems (Figure 6B). It has been reported about the α-glucosidase inhibitory activity of flavonoids (41), and the variability in the results of this study could be due to the difference in the flavonoid contents.

As mentioned previously, caffeoylquinic acid and flavonoids were found to be significantly enriched markers in flowers, suggesting that they may be the main reason for the higher antioxidant activity of this part of the plant. It shows that extracts of *D. nervosa* can be an important source for supply chains in the cosmetic, pharmaceutical and medical industries. In addition, it can be used to produce innovative functional products such as dietary supplements (e.g., coffee supplements), which are receiving a lot of attention for their ability to promote weight loss (42). Roots are the most commonly used ethnobotanical resource and flowers are usually discarded as industrial waste. However, according to research, flowers appear to be a medicinal and functional food with more promising results.

## 4 Conclusion

In summary, this study firstly presented the metabolic profiling of flowers, roots, stems and leaves of *D. nervosa*. A total of 174 non-volatile compounds were identified of various parts by UPLC-Q-Orbitrap-HRMS including hydroxycinnamic acids and derivatives, flavonoids, etc. Forty-five volatile compounds were characterized by GC-MS, including monoterpenes, sesquiterpenes, fatty acids, etc. UPLC-Q-Orbitrap-HRMS and GC-MS combined with multivariate data analysis were able to identify the chemical markers of the samples. It can be seen that there are significant differences in the chemical profile of four parts of the herb. By evaluating the activities of four parts, flowers and roots have the strongest antioxidant activity, while leaves and flowers have exhibited significant α-glucosidase inhibitory activity. This is related to the differences in the metabolites present in different parts. Generally speaking, the difference between different parts can be reflected in the different metabolites detected in the extracts. The identified activity-related chemical markers, which were observed to be concentrated in valuable functional chemical components, can serve as a valid perspective to evaluate the value of different parts of *D. nervosa* (including waste resources like flowers, stems and leaves). Besides, the non-medicinal fraction of *D. nervosa* (especially the flowers) is a larger and cheaper alternative medicinal source than the roots. The present research provides valuable evidence on the molecular basis and pharmacological activities of different parts of *D. nervosa*, facilitating its application in the food chemistry and pharmaceutical industries. However, as the roots have been used as a spice, it is essential to assess the *in vivo* antioxidant and toxicological profiles of other partial extracts as sources of dietary antioxidants for human health, which is an important step in establishing safety limits.

## Data availability statement

The UPLC-Q-Orbitrap HRMS datasets presented in this study can be found in online repositories. The names of the repository can be found at: https://figshare.com/s/172f5b02e8b10b96d714.

## Author contributions

QZ: Methodology, Writing – original draft. YL: Formal analysis, Writing – review & editing. SL: Visualization, Writing – review & editing. XH: Writing – review & editing. RG: Funding acquisition, Resources, Writing – review & editing.
