# Supplementary material for: Comparative bioactivity evaluation and metabolic profiling of different parts of Duhaldea nervosa based on GC-MS and LC-MS
Source: Front Nutr. 2023 Dec 8;10:1301715. doi: 10.3389/fnut.2023.1301715 (PMC10748410; doi:10.3389/fnut.2023.1301715)
Supplement: Supplementary file 2 [file Data_Sheet_1.DOCX]

**Supplementary Materials**

**
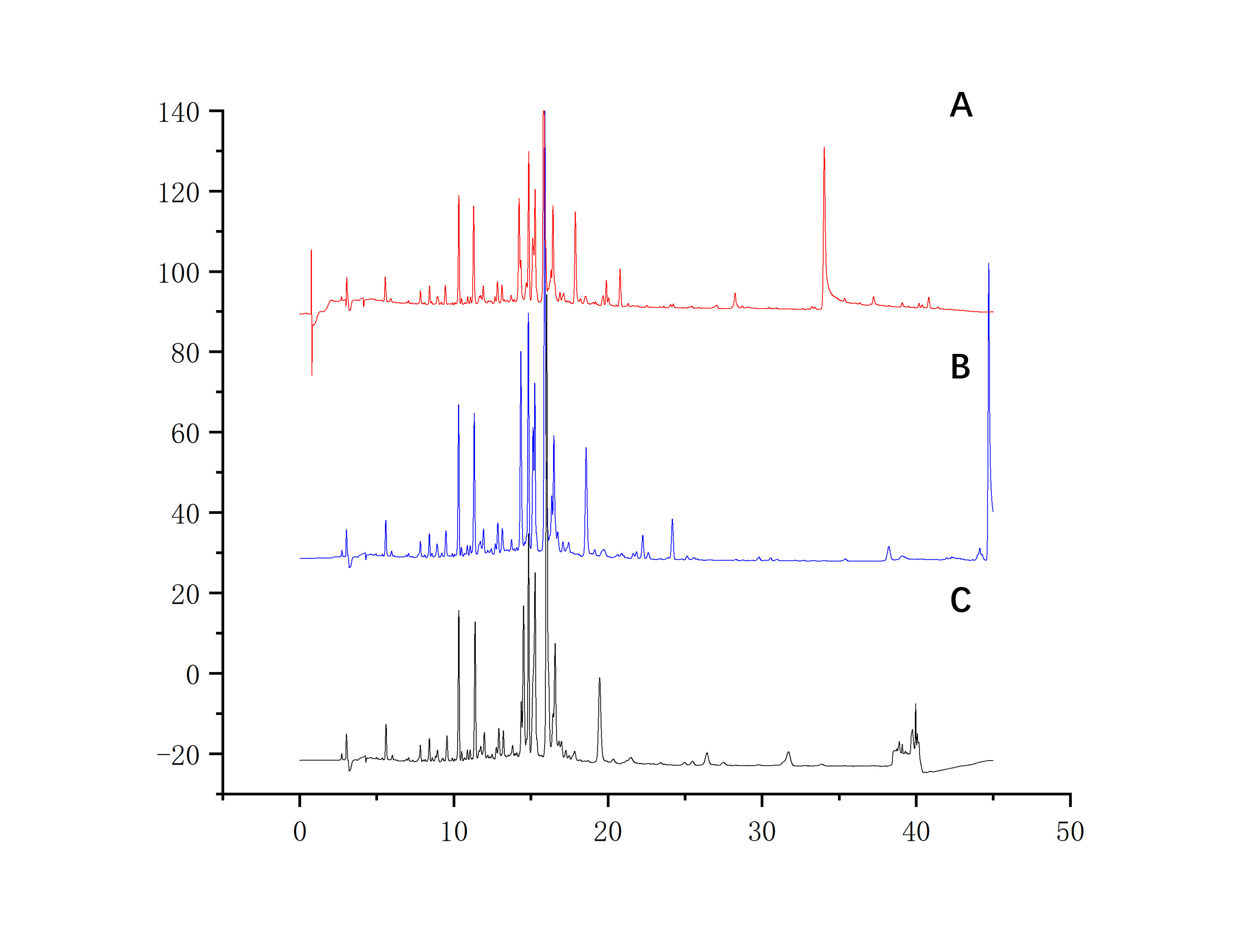
**

**Figure S1.** HPLC chromatograms for three elution conditions of roots at 254 nm; the elution conditions as flowing: **A**. 0-12 2-30% B; 12-35 30-50% B; 25-45 50-95% B. **B.** 0-12 min 2-30% B; 12-30 min 30-32% B; 30-37 min 32-34%B; 37-45 34-95%. **C.** 0-12 min, 2%-30% B; 12-25 min, 30% B; 25-35 min, 30%-32% B; 35-40 min, 32%-34% B; 40-45 min, 34%-70% B; 45-50 min, 70-95% B. According to the HPLC chromatogram, Figure S1-C has a better degree of separation we finally chose condition C as the experimental condition for mass spectrometry


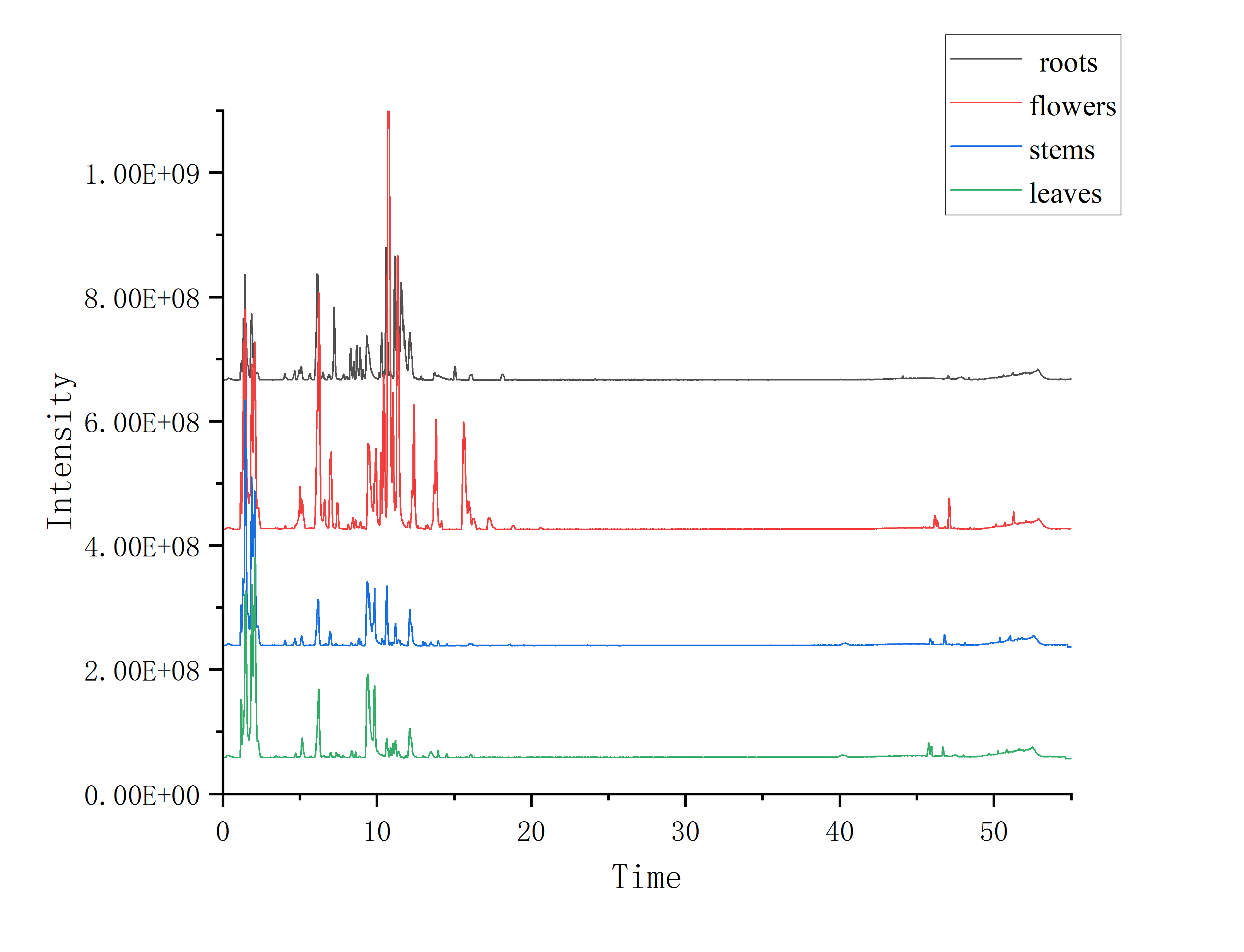


**Figure S2.** UHPLC-Q-Orbitrap-HRMS profiles of different parts in negative ion mode


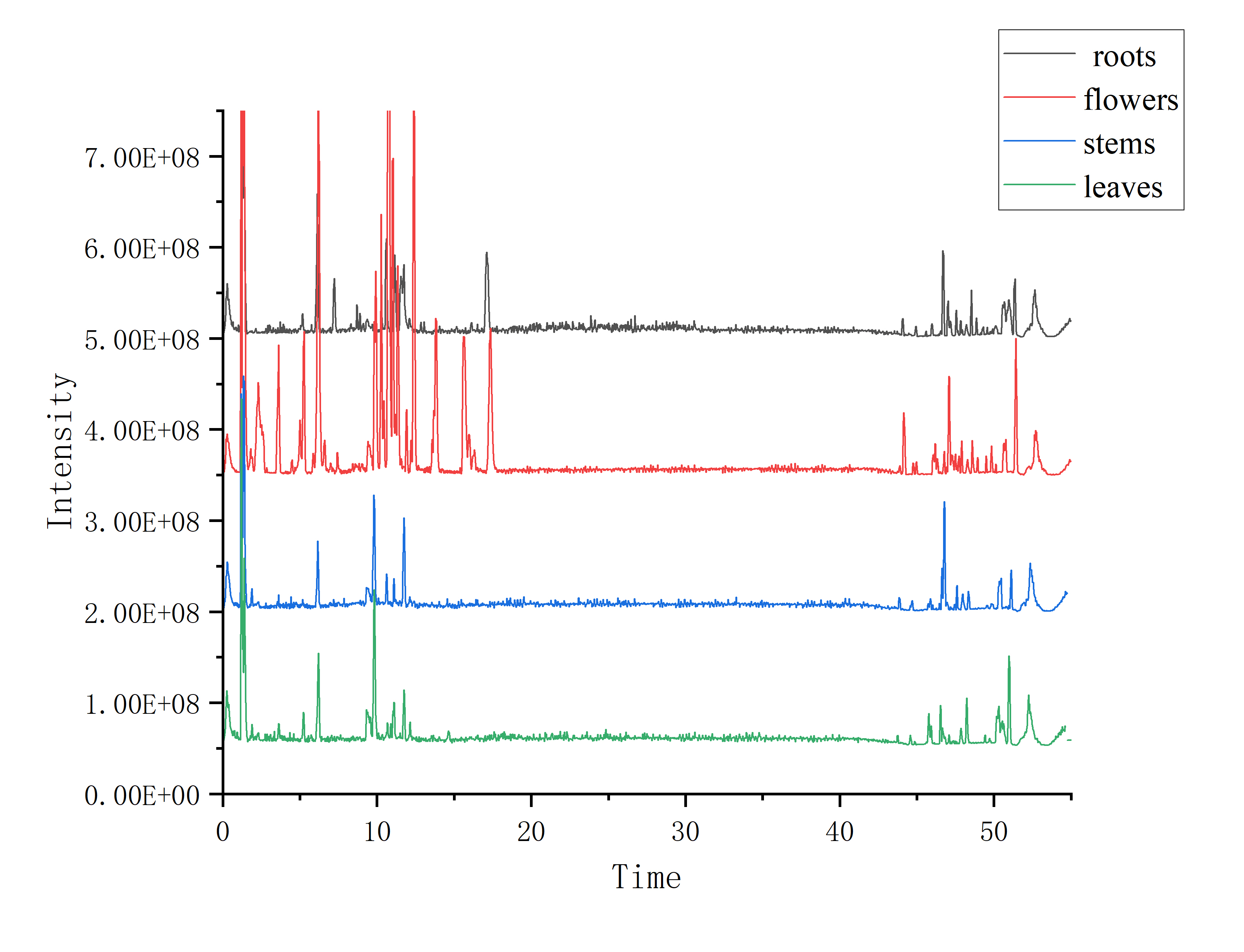


**Figure S3.** UHPLC-Q-Orbitrap-HRMS profiles of different parts in positive ion mode


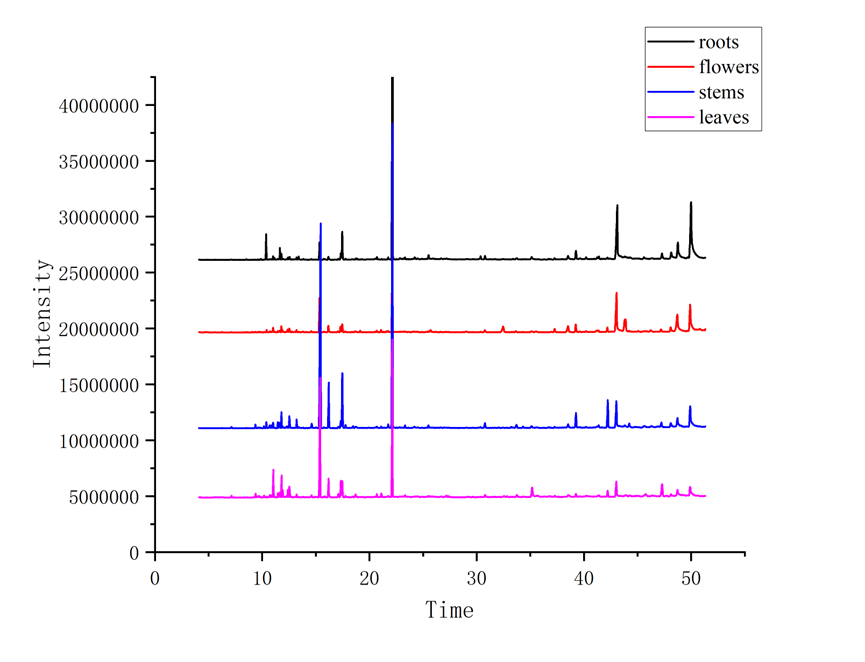


**Figure S4.** GC-MS Profiles of different parts n-hexane extracts of *D. nervosa*
